# History and Current Status of Phytoplasma Diseases in the Middle East

**DOI:** 10.3390/biology10030226

**Published:** 2021-03-15

**Authors:** Chamran Hemmati, Mehrnoosh Nikooei, Ali M. Al-Subhi, Abdullah M. Al-Sadi

**Affiliations:** 1 Department of Plant Sciences, College of Agricultural and Marine Sciences, Sultan Qaboos University, Seeb, Muscat 123, Oman; Chamran.hemmati@gmail.com (C.H.); alsubhia@squ.edu.om (A.M.A.-S.); 2 Minab Higher Education Center, Department of Agriculture, University of Hormozgan, Bandar Abbas 3995, Iran; mehr.nikooei@gmail.com; 3 Plant Protection Research Group, University of Hormozgan, Bandar Abbas 3995, Iran

**Keywords:** Middle East, phytoplasma diseases, insect vectors, 16SrII phytoplasma group

## Abstract

**Simple Summary:**

Phytoplasmas are microorganisms that have been reported to be associated with hundreds of plant diseases in most parts of the world. Several reviews were published regarding diseases associated with phytoplasmas in different countries. However, no comprehensive review is available on the phytoplasma diseases in the Middle East, which is an important region with arid to semi-arid conditions. This review describes the most common phytoplasmas that are associated with diseases in this part of the world. It also describes some of the insect vectors that help to transmit these phytoplasmas. Information is also presented regarding the distribution of the diseases and host ranges.

**Abstract:**

Phytoplasmas that are associated with fruit crops, vegetables, cereal and oilseed crops, trees, ornamental, and weeds are increasing at an alarming rate in the Middle East. Up to now, fourteen 16Sr groups of phytoplasma have been identified in association with more than 164 plant species in this region. Peanut witches’ broom phytoplasma strains (16SrII) are the prevalent group, especially in the south of Iran and Gulf states, and have been found to be associated with 81 host plant species. In addition, phytoplasmas belonging to the 16SrVI, 16SrIX, and 16SrXII groups have been frequently reported from a wide range of crops. On the other hand, phytoplasmas belonging to 16SrIV, 16SrV, 16SrX, 16SrXI, 16SrXIV, and 16SrXXIX groups have limited geographical distribution and host range. Twenty-two insect vectors have been reported as putative phytoplasma vectors in the Middle East, of which *Orosius albicinctus* can transmit diverse phytoplasma strains. Almond witches’ broom, tomato big bud, lime witches’ broom, and alfalfa witches’ broom are known as the most destructive diseases. The review summarizes phytoplasma diseases in the Middle East, with specific emphasis on the occurrence, host range, and transmission of the most common phytoplasma groups.

## 1. Introduction

The Middle East is a transcontinental region that includes Western Asia, Egypt, Iran, and Turkey, and in which agriculture plays a vital economical role. The wide range of temperature fluctuation makes it possible to cultivate a diverse variety of crops, including fruits, vegetables, nuts, cereals, tea, tobacco, and medicinal herbs. In the Middle East, more than 20,000 plant species are grown [1]. Date palm is one of the most important crops in this part of the world. It is widely cultivated in most countries of the Middle East [2]. Among the ten top producers of dates in the world, six countries are from the Middle East (Egypt, Saudi Arabia, Iran, Iraq, Oman, and UAE) [2]. Other important crops include wheat, tomatoes, potatoes, sugarcane, maize, sugar beet, and citrus. 

**Figure 1:**
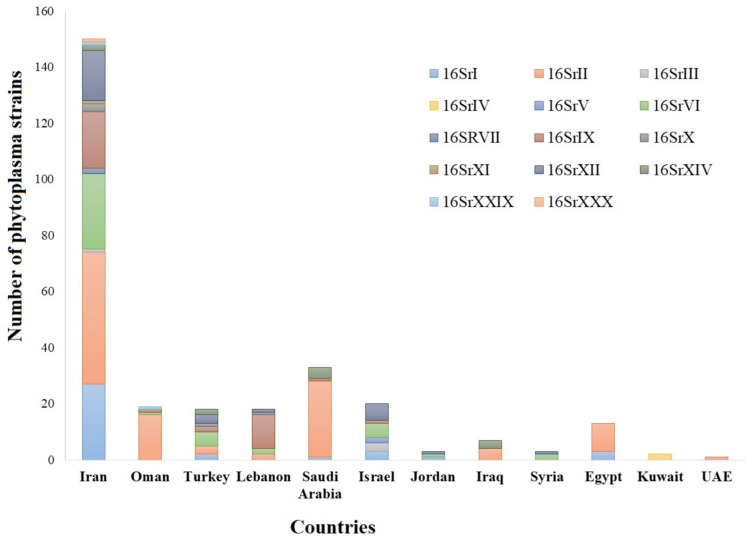
Distribution of phytoplasma groups in countries of the Middle East. The colors represent phytoplasma groups, while the numbers indicate the number of hosts infected by phytoplasmas from each group.

**Figure 2:**
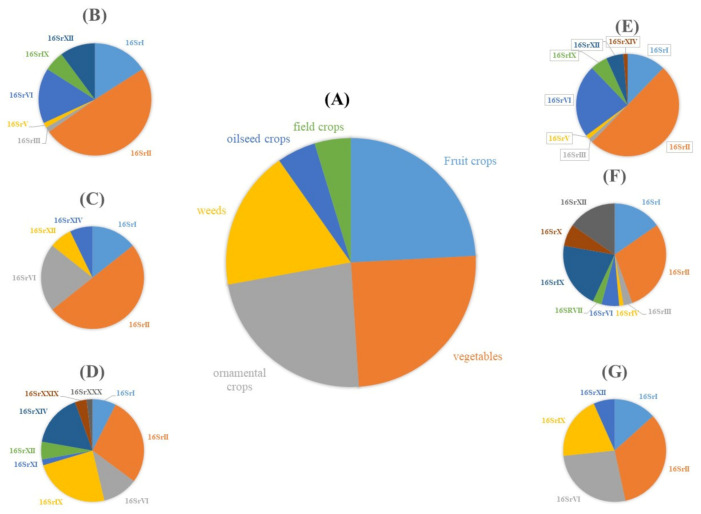
Distribution of phytoplasma groups in different crops, where (**A**) shows the relative occurrence (%) of phytoplasma diseases according to hosts, while the lateral pies show the relative occurrence (%) of phytoplasma groups on in ornamental crops (**B**), field crops (**C**), weeds (**D**), vegetable crops (**E**), fruit crops (**F**), and oilseed crops (**G**).

Many plant diseases that are associated with fungi, phytoplasmas, nematodes, viruses, and viroids have been reported in the Middle East [3,4,5,6,7]. Most plant diseases in the Middle East are caused by fungal pathogens [8]. However, the most challenging diseases have been reported as a result of phytoplasma or bacterial diseases [9,10]. Phytoplasmas have been reported in 164 plant species, including vegetables, cereals, fruit crops, medicinal herbs, shade trees, and forage crops. Among the 34 phytoplasma ribosomal groups reported globally, 14 were reported from the Middle Eastern countries (Figure 1 and Figure 2). 

Phytoplasmas, which belong to the Mollicutes class, are wall-less pleomorphic phytopathogenic bacteria, annually destroying many economical plant species worldwide [11]. They have a diameter of 200–800 nm [12] and they are found only in the phloem of vascular plants and in the gut and salivary glands of some sap-sucking insects [13,14,15,16]. Phytoplasmas’ membranes usually contain three types of immunodominant protein (IDP): immunodominant membrane protein (Imp), immunodominant membrane protein A (IdpA), and antigenic membrane protein (Amp) [17]. Some IDPs play a role in host–phytoplasma interactions [18]. The Amps interact with leafhoppers microfilament complexes, thus playing a role in determining the specificity of insect vectors to phytoplasmas [19,20,21]. Phytoplasmas can modulate and regulate plant hosts genes, hormones, and secondary metabolite biosynthesis [22]. Phytoplasmas produce proteins, called effectors, which help them overcome plant defenses [23,24]. Effectors help phytoplasmas to multiply in plant hosts, spread by insects, and to modulate plant host growth. Additionally, phytoplasmas secrete effector proteins that alter plant development and enhance plant susceptibility to the insect vectors [14,25]. 

Phytoplasmas can induce disease symptoms, such as witches’ broom, phyllody, reddening and yellowing of leaves, virescence, proliferation of the shoots, and generalized stunting [26]. Some conserved genes, including 16Sr, ribosomal protein (*rp*), elongation factor TU (*tuf*), and translocase protein A and Y, have been utilized to classify them into diverse ribosomal groups and subgroups [27]. To date, 41 ‘*Candidatus*’ species, 33 ribosomal groups, and 160 subgroups were categorized based on RFLP analyses and/or sequencing of the 16S rDNA [28].

The feeding behavior of insect vectors plays an important role in the geographical distribution of phytoplasma strains [29]. In addition, transportation and the use of infected plant material for grafting and planting can result in the spread of phytoplasma diseases [9,30,31]. For example, the 16SrI group phytoplasma strains are transmitted by over 30 species of insect vectors, most of them polyphagous, and they can infect over 100 plant species worldwide, resulting in its vast geographical distribution [28]. On the contrary, phytoplasmas belonging to the coconut lethal yellowing (16SrIV), ash yellows (16SrVII), and apple proliferation (16SrX) groups are distributed in restricted regions and they are transmitted by specific vectors [32,33,34,35]. Plant disease epidemiologists are concerned about the impact of climate change on insect life cycle, as vectors might become more active and introduce phytoplasmas into new areas, hence expanding their geographical distribution [36]. In addition, climate change also has an impact on plants that are grown in a specific area, as it can restrict growth of certain plant species or allow for the introduction or widespread cultivation of new or less common plant species [37]. Given that quarantine measures are not strict in many countries in the Middle East [38,39], the frequent import of ornamental plants and crops from different countries has been associated with the introduction of new pathogens and strains into these countries [7,40]. Although phytoplasmas can evolve to attack new crops [41], limited studies addressed phytoplasma strain evolution in this part of the world. Therefore, data information on epidemic phytoplasmas, their associated host plants, and their vectors in the Middle East are important for quarantine purposes. 

To our knowledge, there is no published review on phytoplasma diseases in the Middle East, except for the article that was written by Siampour et al. [42] about phytoplasma diseases occurring in Iran. When considering the high economic importance of phytoplasma diseases, we collected all available publications regarding phytoplasma diseases occurrence and epidemiology in the Middle East since the early 1970s until the present time. This review presents the associated hosts, phylogenetic relationships, and the insect vectors of the identified phytoplasmas in the Middle East. Given the high number of new phytoplasma strains and associated diseases reported on a monthly basis in the Middle Eastern countries, this review will be a reference for everyone looking for information about phytoplasma occurrence and epidemiology in this part of the world.

## 2. Survey Methodology

A survey was done in Scopus (www.scopus.com, accessed on 25 November 2020), web of science (www.webofknoledge.com, accessed on 20 December 2020) and google scholar (www.scholar.google.com, accessed on 13 July 2020). The access dates in these websites were during 10 January 2020 and 17 April 2020. Most of the literature was obtained through Scopus (>90%). The survey focused on all articles reporting phytoplasma occurrence and associated diseases in the Middle East. The search terms included, but were not limited to, phytoplasma, Middle East, witches’ broom, and other phytoplasma related symptoms. Reports from countries other than the ones located in the Middle East were excluded after reading the abstracts or papers. In addition to the search engines, we also relied on reports that were submitted by researchers to the relevant governmental institutions (e.g., Ministry of Agricultural, Marine and Water resources, Oman). This ensured coverage of most literature that dealt with phytoplasma diseases in this part of the world.

## 3. A Historical Overview of Phytoplasma Diseases in the Middle East

Phytoplasmas are associated with diseases in hundreds of plant species worldwide [29,43,44]. Losses due to phytoplasmas range from negligible effects on the growth to a complete decline of infected plants. Lethal yellowing diseases (LYD) of palms resulted in the death of millions of palms, especially in the Caribbean and Africa [45]. In Europe, apple proliferation, pear decline, and European stone fruit yellows (ESFY) diseases resulted in substantial losses [46]. In China and India, ‘*Cadidatus* Phytoplasma ziziphi’ phytoplasma has been associated with severe outbreaks and losses in jujube, cherry, and peach [47]. Rice orange leaf (ROL) and rice yellow dwarf (RYD) diseases are some of the other common phytoplasma diseases in Asia [48,49].

In the Middle East, several phytoplasma diseases are widespread. Some of the diseases only cause significant losses in this part of the world, such as witches’ broom disease of acid lime [50,51], while others are found to be associated with diseases in other parts of the world, such as phytoplasma diseases on almond, stone fruits, tomatoes, carrots, etc. [52,53].

The first observation of a phytoplasma disease in the Middle East dates back to the early 1970s, when witches’ broom disease symptoms were observed on acid lime (*Citrus aurantifolia*) in the northern parts of Oman [54,55]. Because they could not confirm the causal agent, a subsequent survey in the early 1980s by J.M. Bové showed that mycoplasma-like organisms (MLO, former name for phytoplasmas) were associated with the disease [56]. In 1987, symptoms of severe witches’ broom were observed on alfalfa in Kerman province in Iran by Rahimian [57]. Witches’ broom of acid lime was reported in the United Arab Emirates in 1989 [58]. No molecular work was done to identify and characterize the associated phytoplasma until 1993 [59]. The first phytoplasma reported in Iran was associated with sesame phyllody [60]. In Israel, research on phytoplasmas started in the 1980s on grapevine, although phytoplasma symptoms have been observed in the 1970s on Bermuda grass and strawberry [61]. In Iraq, the first observation dates back to 2015, when phytoplasma that was associated with Arabic Jasmin was characterized [62]. In Lebanon, a destructive phytoplasma disease on almond was characterized in the early 2000s [63]. In Egypt, the first report of phytoplasma disease was during 2001 with the description of phytoplasma disease symptoms associated with mango [64]. In Saudi Arabia, the first record of a phytoplasma disease was published in 2007 with the description of Al-Wijam, a phytoplasma disease occurring on date palm and associated with 16SrII [65]. 

Nowadays, many phytoplasma diseases are being reported from countries in the Middle East. The advances in sequence technology and molecular identification provided by PCR helped to detect 14 16Sr groups of phytoplasma that are associated with 164 plant species (Table S1).

## 4. Phytoplasmas Associated with Fruit Crops

Fruit crops are widely grown in the Middle East, with date palms being the most important fruit crop. Other fruit crops include *Citrus* spp., *Prunus* spp., grapevines, pomegranates, pears, apples, and bananas.

### 4.1. Prunus Species

The genus *Prunus* includes 430 plant species and it is distributed through temperate regions of the world. Nectarine, peach, European plum, almond, Japanese plum, sour cherry, apricot, and sweet cherry are known to be the most economically important *Prunus* species [28]. Several phytoplasma groups threaten *Prunus* production. The strains of the 16SrI-B, 16SrII (subgroup II-B, II-C), 16SrVI (subgroup VI-A and VI-D), 16SrIX (IX-B, IX-C, IX-D), 16SrX-F, and 16SrXII-A are the major phytoplasma groups causing economic losses in *Prunus* species in the Middle East [66,67,68].

Almond witches’ broom (AlmWB) is a destructive disease of stone fruits in Lebanon and Iran [69]. In 2018, Iran was the main almond producer among the Middle Eastern countries, with 139,000 tons of yearly almond production, followed by Turkey (100,000 tons/year) [2]. Witches’-broom of almond (AlmWB) was first observed in Lebanon in the early 1990s and then in Iran in 1995 [63,70,71]. In Iran, AlmWB phytoplasma was primarily reported from almond orchards in the center of Iran and then identified in association with peach and almonds in other regions in Iran [72]. In Lebanon, it has been estimated that this disease destroyed more than 100,000 trees of almond during the outbreak that occurred in 2002, which was attributed to a new strain of phytoplasma [71,73]. The disease was also reported on peach and nectarine in Lebanon and Iran [69,74]. 

Abou-Jawdah, Karakashian, Sobh, Martini, and Lee [71] revealed that a strain of pigeon pea witches’ broom group was associated with AlmWB in Lebanon. Further studies showed that two different strains, 16SrIX-C and 16SrIX-B, were also associated with AlmWB; the latter was identified as a novel taxon and named ‘*Ca*. P. phoenicium’ [11,74]. Both of the subgroups are associated with AlmWB in Iran, while only the 16SrIX-B strain is known as the causal agent of AlmWB in Lebanon [73]. It has been found that all stone fruit trees were only associated with 16SrIX-B in Lebanon and Iran, although phytoplasmas belonging to the 16SrIX-C group are also known to be associated with wild plant species in Lebanon [74]. Abbasi, Hasanzadeh, Zamharir and Tohidfar [66] revealed that, in Iran, sweet orange can be infected by the 16SrIX-B phytoplasma. In addition, further studies revealed that the natural hosts of this strain in Iran include peach, apricot, nectarine, wild almond, *Smalix aspera*, and *Anthemis* spp. [75,76]. It has also been reported that other phytoplasma groups, including 16SrII-C, 16SrVI-D, and 16SrVII-A, were associated with ALmWB in Iran [77,78].

The 16SrIX-B and 16SrIX-C induce similar symptoms after 1–2 years of infection, including the proliferation of the auxillary branches, leaf yellowing, witches’ broom, severe dieback, the decline of the tree, and loss of production [74]. The main symptoms on nectarine and peach include early bud emergence, little leaves and yellowing, witches’ broom, and phyllody [79]. Zirak, et al. [80] reported that Japanese plum and cherry grown in Iran were associated with aster yellows (16SrI), peanut witches’ broom (16SrII), and stolbur phytoplasma group (16SrXII). Symptoms included yellowing, witches’ broom, proliferation of the shoots, and little leaf [52].

*Prunus* species can also be affected by another phytoplasma disease, namely European stone fruit yellows (ESFY). Plum leptonecrosis, apricot chloritic leaf roll, decline of peach, and plum yellowing are the disease symptoms that are caused by ESFY [81]. The phytoplasmas that are associated with ESFY are strains of ‘*Ca*. P. pronurum’ (16SrX-B) [82]. However, strains related to pigeon witches’ broom phytoplasma (16SrIX) have also been reported to be associated with *P. scoparia*, *P. persica*, and *P. armeniaca* in Iran [77,83]. In Israel, the association between 16SrXII-A and apricot (*P. armeniaca*) has also been reported [84]. In addition, studies revealed that sweet cherry (*P. avium*) was associated with ‘*Ca*. P. asteris’ in Iran and Turkey [85], ‘*Ca*. P. aurantifolia’ in Iran [68], and ‘*Ca*. P. trifolii’ in Israel [86]. Phytoplasma strains of 16SrII-B, X-F and XII-A have been reported to be associated with *P*. *domestica* in Iran [68,83]. The association between *P. persica* and ‘*Ca*. P. trifolii’ and 16SrI has been reported in Iran and Jordan, respectively, although the phytoplasma subgroup was not identified in Jordan [87]. 

Studies that were conducted in Lebanon revealed that the polyphagous leafhopper *Asymmetrasca decedens* is the vector of 16SrIX-B in Lebanon [88]. In addition, this leafhopper has been reported from almond orchards in Iran [89]. Transmission trials by *Tachycixius viperinus* Dlabola and *T. cypricus* Dlabola revealed that they can transmit the 16SrIX-B phytoplasma to peach in Lebanon [90]. *T. viperinus* and *T. cypricus*, collected from *Anthemis* spp. and *Smilax aspera*, which were grown in the wild in Lebanon, also tested positive to AlmWB phytoplasma strains [90]. However, the vector of AlmWB disease has not been identified in Iran [73,80].

### 4.2. Pear

Pear decline (PD) is considered to be one of the most destructive pear diseases in the world. ‘*Candidatus* Phytoplasma pyri’ (16SrX-C) is the causal agent of PD and it has been reported in Lebanon and Iran [91]. The typical symptoms of PD include reddening and yellowing of the leaves, stunting, and abnormal foliage [82]. It has been shown that the associated phytoplasma overwinters in the root, so the rootstock cultivar can play a role in the severity of the symptoms. ‘*C.* P. pyri’ (16SrX-C) is the phytoplasma strain that is associated with PD in Iran and Lebanon [91]. In addition, the 16SrI and 16SrX-B phytoplasmas have been reported to be associated with pear decline in Iran [82].

*Cacopsylla pyricola* has been confirmed as a phytoplasma vector in Europe and the USA [92]. Although this species has been detected in Iran, no information is available regarding its ability to spread PD phytoplasma. 

### 4.3. Apple

A phytoplasma disease that is similar to apple proliferation (AP) has been observed in apple orchards in Turkey, Lebanon, and Iran. The symptoms included defoliation, yellowing of leaves, enlarged stipules proliferation, small fruits, several small shoots, and decline. Phytoplasma strains that are associated with AP belong to the 16SrI and 16SrII groups in Iran [93], 16SrX-A in Turkey [94], and 16SrIX-C in Lebanon [95]. In addition, an association between 16SrIX-C and a wild apple *Malus sylvestris* exhibiting virescence symptoms has been reported in Lebanon, which suggests that wild apple can work as a reservoir of AP and facilitates its spread [95]. However, little information is available on the relationship between these phytoplasmas strains and apple cultivars.

### 4.4. Grapevine

Grapevine (*Vitis vinifera* L.) is common in Iran, Turkey, Jordan, and Syria. The most economical phytoplasma disease of grapevine is Flavescence dorée (FD), which is associated with 16SrV-C and 16SrV-D subgroup. This disease is prevalent in many grape-growing areas in Europe, but it has not been detected in the Middle East [96]. Grapevine is known to be a host for several phytoplasmas that cause grapevine yellow (GY). The symptoms of GY include reddening and yellowing in red and white cultivars, necrosis of terminal buds, the appearance of black pustules in infected shoots, downward curling of leaves, and the decline and drying of the berries [97]. Seven phytoplasma groups have been reported associated with GY symptomatic grapevine plants, including the 16SrII-B subgroup in Iran [98], 16SrVI in Syria [99], 16SrI, 16SrIII, and 16SrXII in Israel [61], and 16SrI-B, and 16SrIX in Turkey [100]. 

Bois noir (BN), which is associated with stolbur phytoplasma strains (subgroup 16SrXII-A, ‘*Ca*. P. solani’), is a widespread and significant phytoplasma disease in grapevine which was reported from Iran [101], Turkey [100], and Jordan [102]. It has been confirmed that *Hyalesthes obsoletus* is the vector of BN in Europe [103] and, although its presence has been confirmed in vineyards grown in Iran, its ability to transmit BN phytoplasma to healthy plants has not been tested. 

Other studies revealed that other phytoplasma groups, such as 16SrI (‘*Ca*. P. asteris’), 16SrVII (ash yellows, ‘*Ca*. P. fraxini’), and 16SrIX-C (‘*Ca*. P. phoenicium’), have been detected in symptomatic grapevines in the center and south of Iran [98,104,105].

The leafhopper *Hyalestes obsoletus*, and *Circulifer orientalis* are known as vectors of grapevine yellows in Israel [106]. 

### 4.5. Date Palms

Date palm (*Phoenix dactylifera* L.), which is one of the oldest known fruit crops in the Middle East, is the most important fruit crop in Middle East, North Africa, and Arabian Peninsula. Based on FAO statistics, date palm production reached 8.5 Mt in 2018, with Egypt, Saudi Arabia, and Iran being the top producers [2]. The association between date palm and phytoplasma groups 16SrI in Egypt [107], 16SrII in Saudi Arabia [108], and 16SrIV in Kuwait [109] have been documented. The latter is the only record of the 16SrIV group in the Middle East. Recently, the association between 16SrVI and 16SrVII and date palms has been reported in Iran [110], while the 16SrII-D subgroup has been reported to be associated with date palm streak yellows in Oman [111].

### 4.6. Acid Lime

Witches’ broom disease of lime (WBDL), the most devastating phytoplasma disease infecting Mexican limes, was first observed in Oman in the early 1970s [55]. This disease was then observed in the UAE in 1989 and destroyed many lime orchards in both countries [58]. WBDL was reported in Iran in 1997 and it has been reported in Saudi Arabia in 2009 [112,113]. The infected trees develop a large number of small branches and leaves. The leaves are smaller in size and light green to yellow in color. The symptomatic branches do not usually produce fruits and the infected trees usually die withing four to eight years after the first appearance of symptoms [114]. The disease killed over one million lime trees in Oman and Iran [51].

Other citrus species, like grapefruit, citron, limequat, *Citrus macrophylla*, *C. limonia*, *C. jambhiri*, and, especially, Bakraee, have been reported as hosts of the WBDL phytoplasma in Iran [66,115,116]. In Oman and the UAE, WBDL also infects Palestine sweet lime, citron, *Citrus macrophylla*, and sweet limetta [117]. WBDL symptoms were rarely observed on grapefruits [118,119].

*‘Ca.* P. aurantifolia’, the only member of the 16SrII-B subgroup, is the causal agent for the WBDL disease in Iran as well as in Oman, Saudi Arabia, and UAE [120,121]. 

In acid lime, the rapid spread of this disease in the infected areas reinforced a hypothesis of involvement of an insect vector. The successful transmission of the phytoplasma by *Hishimonus phycitis* to Bakraee seedlings was reported by Salehi, Izadpanah, Siampour, Bagheri, and Faghihi [118]. Subsequently, Bagheri, Salehiz, Faghihi, Samavi, and Sadeghi [119] collected 1000 *H. phycitis* from WBD-affected limes, which were transferred to four healthy acid lime trees (the absence of phytoplasma confirmed by PCR) and covered with an insect-proof net. Three trees developed WBDL symptoms as compared to no symptoms in control trees exposed to 500 *H. phycitis* that were collected from disease-free fields. Queiroz, et al. [122] reported that the Asian psyllid *Diaphorina citri* could also transmit phytoplasma to healthy plants with an efficiency 20 times lower when compared to *H. phycitis*. Hemmati, et al. also confirmed the transmission of phytoplasma to Mexican lime seedlings by *H. phycitis* [123]. 

### 4.7. Pistachios and Other Fruit Crops

Pistachios (*Pistacia palaestina*) is one of the most important fruit crops in Iran, which are mainly exported to other countries. To date, phytoplasmas belonging to the subgroups 16SrII, 16SrIX, and 16SrXII-A have been reported to be associated with pistachio yellows in Iran [124]. Casati et al. (2016) reported that *P. palaestina* was affected by 16SrIX-C in Lebanon. Symptoms on pistachios include severe witches’ broom, stunted growth, yellowing, and malformation [124]. 

Other fruit crops were also reported to be associated with phytoplasma. For example, Salehi, et al. [125] reported that pomegranate (*Punica granatum*) was associated with ‘*Ca*. P. australasia’ and ‘*Ca*. P. pruni’ in central and northeast Iran and phytoplasma belonging to subgroups 16SrII-B and 16SrII-C were reported in Chicoo and Barberry in Iran, respectively [126,127]. A destructive disease, named Nivun Haamir dieback (NHDB), has been reported in association with 16SrXII-A subgroup on papaya (*Carica papaya*) in Israel [128].

## 5. Phytoplasmas Associated with Cereal and Forage Crops 

### 5.1. Cereal Crops

Cereals are staple food in all parts of the world. The most cultivated cereals are wheat, rice, rye, barley, corn, and sorghum. Maize was found to be associated with the 16SrVI-H in Iran and 16SrXIV-A in Turkey [129]. In addition, phytoplasmas belonging to subgroup 16SrVI-A were found in sorghum plants that were grown in northwest of Iran [130].

### 5.2. Forage Crops

The most known phytoplasma disease of sugarcane is white leaf (SCWL), which is associated with 16SrII strains in Iran [42] and 16SrI strains in Egypt [131]. The stunting of the infected plants, leaf blades with stripped white color, and frail leaf and death of plants are the symptoms of SCWL. 

Most countries in the Middle East cultivate Alfalfa (*Medicago sativa* L.). The most common disease of alfalfa is alfalfa witches’ broom (AlfWB), which has been reported in several countries. Several phytoplasma groups, including 16SrXII, 16SrVI, 16SrII, and 16SrI, have been reported in association with this disease in the Middle East. The symptoms of the disease include little leaves, stunting, witches’ broom, decline, and plant death [57,132]. Phytoplasma strains of the 16SrII group are the major causal agents of AlfWB in the Middle East. Indeed, only phytoplasma strains of the 16SrII-D subgroup were associated with AlfWB in Oman, Iraq, and Saudi Arabia [132,133,134]. However, 16SrXII has been reported from several alfalfa fields in Iran [135]. Esmailzadeh-Hosseini, et al. [136] confirmed that *O. albicinctus* is the vector of the 16SrII phytoplasmas causing AlfWB in Iran. In addition, weed species, including *Cardaria draba* and *Prosopis fracta*, known preferred hosts for vectors, have also been identified as alternative hosts of the 16SrII phytoplasmas causing AlfWB [57].

## 6. Phytoplasmas Associated with Vegetable Crops

Vegetables are widely grown in the Middle East. Several vegetable crops have been introduced into countries in the Middle East during the last 50 years. The production and area of cultivation of vegetable crops increased dramatically over the last years, making them an important source of income for growers (FAO 2019). To be more specific, tomato and watermelon production increased by 1500% and 500%, respectively, over the last 40 years in Oman (FAO 2019).

Phytoplasmas cause important diseases in vegetable crops. Phytoplasma diseases have been reported in cucumber, carrot, tomato, potato, faba bean, lettuce squash, parsley, pepper, onion, spinach, and cabbage, where witches’ broom, big bud, and phyllody are among the most commonly described symptoms observed on vegetables [137,138]. 

### 6.1. Solanaceae

Among the vegetable crops in the Middle East, tomato is the host for many phytoplasmas groups. Tomato big bud (TBB) is the most important tomato disease, which was found to be associated with the 16SrI, 16SrII, 16SrVI, 16SrIX, and 16SrXII phytoplasmas in Iran [139]. In Turkey, TBB was associated with 16SrVI-A and 16SrVII-A [140]. In addition, a strain of 16SrVI was associated with tomato big bud disease in Jordan [141] and Syria [142]. TBB symptoms include phyllody, virescence, enlarged and changed calyxes to leaf-like, the proliferation of the axillary shoots, and witches’ broom [139].

Tomato witches’ broom (ToWB) associated with 16SrII-D has been reported in the south of Iran. In addition, phytoplasmas belonging to the 16SrII-D subgroup have been identified in tomato grown in Oman [139], Iraq [62], Egypt [143], and Saudi Arabia [144]. Moreover, a phytoplasma of the 16SrVI group has been reported in association with this disease in Lebanon [145]. ToWB can be easily diagnosed by visual inspection of symptoms, which include witches’ broom, stunting, small deformed leaves, and the proliferation and lack of flowers. Omar and Foissac (2012) reported that *Empoasca decipiens* is the vector of tomato witches’ broom disease in Egypt.

Other important phytoplasmas on Solanaceae crops include the 16SrII-D on eggplant in Egypt [146], the 16SrII-B and 16SrVI-A on potatoes in Jordan [147] and Lebanon [145], respectively, and the 16SrII-X on potato and eggplant in Saudi Arabia [148]. The phytoplasma groups that are associated with phytoplasma vegetable diseases in Turkey and Israel are different. For example, pepper with stolbur symptoms was found to be associated with 16SrVI-A in Turkey [140], while it was found to be associated with 16SrXII-A in Israel [86]. The difference could be because phytoplasmas originated from different sources or that different vectors exist in both countries [86]. In Iraq, eggplant was reported to be the host of a phytoplasma in the 16SrII-D subgroup [62].

### 6.2. Cucurbits

Several 16SrII phytoplasma strains were found to be associated with cucurbits. For example, squash and cucumber phyllody were associated with strains of the 16SrII group in Iran [149]. In some countries, it has been reported that the phyllody of cucurbitaceous crops can result in 100% crop loss in the case of early infection [137]. In Egypt, the association between 16SrII-D and squash has been reported [146]. ‘*Ca.* P. australasia’ was reported on squash in Oman [138]. 

### 6.3. Lettuce

Phyllody of lettuce (*Lactuca sative* L.) and wild lettuce (*Lactuca serricola* L.) has been reported in Iran and it can severely affect lettuce production [150,151]. Phytoplasma strains that are associated with both diseases belong to the 16SrI (‘*Ca*. P. asteris’) and 16SrIX (‘*Ca*. P. phoenicium’) groups, subgroup B. The symptoms include the proliferation of the buds in the crown, deformed chlorotic and small leaves, and stunting and death of the infected plants. 

### 6.4. Carrot

Carrots grown in Iran and Israel have been reported to host phytoplasma groups causing carrot yellows (CY) [152,153]. The phytoplasma groups that are associated with CY are members of the 16SrI, 16SrIII, and 16SrV groups [153]. This disease induces symptoms such as the little leaf, witches’ broom, opening of the head and stunting. In Israel, it has been reported that *Circulufer haematoceps* and *Neoaliturus fenestratus* are the putative vectors of these phytoplasmas [153]. 

## 7. Other Vegetable Crops

Phytoplasma diseases on peas have also been reported in Iran and Oman. For example, the 16SrIV phytoplasma was reported to be associated with *Phaseolus vulgaris* and *Glycin max* in Iran [154], and the 16SrII-D was reported on *Cicer arietinum* in Oman [155]. Faba bean phyllody was associated with strains of the 16SrII group in Iran [149]. ‘*Ca.* P. australasia’ strains are more common on faba bean in Oman [138].

Sugar beet (*Beta vulgaris*) has been reported to be a host for phytoplasma strains belonging to the 16SrII group. In Iran, 16SrII-E was associated with *B. vulgaris* [156], while 16SrII-X was also reported in Saudi Arabia in *B. vulgaris* [149]. Onion, faba bean, and eggplant were identified as the hosts of 16SrII-D in Saudi Arabia [157,158]. The association between 16SrVI-D and red cabbage was also reported in Iran. In addition, cabbage showing multiple heads and deformation of heads was associated with 16SrII-D in Saudi Arabia and Oman [138,148]. 

*Orosius albicinctus* was identified as a vector of carrot witches’ broom and squash and cucumber phyllody in Iran [152,159]. *O. albicinctus* can possibly play a significant role in the spread of 16SrII phytoplasmas infecting vegetables due to the ability to transmit diverse phytoplasma groups and because of its wide distribution in Iran [159,160]. Among several leafhoppers that were collected from infected fields, only *C. haematoceps* could successfully transmit the 16SrII phytoplasmas to healthy carrot, rapeseed, cauliflower, and periwinkle [152]. Salehi et al. (2007) stated that *N. fenestratus* can transmit these phytoplasma strains to healthy lettuce plants as well as periwinkle and sow thistle [150]. 

## 8. Phytoplasmas Associated with Oilseed Crops

Oilseed crops are important to the economy of several countries in the Middle East. The most common oilseed crops in these countries include sesame, rapeseed, sunflower, and safflower [2].

### 8.1. Sesame

The association between different groups of phytoplasmas and oilseed crops has been reported in the Middle East. Phytoplasma strains of peanut witches’ broom, clover proliferation, and pigeon pea witches’ broom have been found to be in association with sesame phyllody (SP) in Iran, Oman, and Turkey [140,159,161]. The symptoms of SP include flower sterility, little leaf, virescence, phyllody, witches’ broom, and stunting. Phytoplasmas belonging to the 16SrII-A, II-D, 16SrVI-A, and 16SrIX-C groups were found to be associated with sesame phyllody in Iran and Turkey [159]; however, an association between 16SrII-D and sesame was reported in Oman. Sesame phyllody was also reported in Syria and Egypt [162,163]. 

Salehi et al. (2017) revealed that the leafhopper *Circulifer haematoceps* can vector all the phytoplasma strains (16SrII-A, II-D, 16SrVI-A, and 16SrIX-C) that are associated with SP in Iran as opposite to *O. albicinctus*, which only transmitted the 16SrII-D phytoplasma. However, it has been confirmed that *O. albicinctus* is able to transmit 16SrIX-C phytoplasma strains in Syria and Turkey [162]. 

### 8.2. Rapeseed

Rapeseed is another important oilseed crop that is cultivated around the world. To our knowledge, Iran is the only country in the Middle East where a phytoplasma disease was reported in rapeseed. Infected plants showed proliferation, witches’ broom, floral sterility, virescence, and phyllody. Sequence analysis showed that a 16SrI-B strain was associated with rapeseed phyllody in Iran [164]. 

### 8.3. Asteraceae

An association between safflower and 16SrVI-C has been reported in Iran. Phytoplasma induced virescence, floral sterility, phyllody, proliferation, and little leaf symptoms in the infected plants [165]. Sunflower phyllody include symptoms of head abnormality, virescence, proliferation, phyllody, and it has been associated with 16SrII-D in Iran. The 16SrVI group has been also detected with 16SII-D coinfecting sunflower [166,167]. It has been reported that strains of 16SrI group were associated with niger seed phyllody and canola in Iran [151,166]. 

## 9. Phytoplasmas Associated with Ornamentals, Weeds and Rangeland Plants

The Middle East contains several native plants that are of several uses, including their use as ornamental plants or for medicinal purposes [168]. Several ornamentals and weeds have been identified as hosts of phytoplasma groups from diverse geographical regions in the Middle East. So far, five phytoplasma groups, including 16SrI, 16SrII, 16SrVI, 16SrIX, and 16SrXII, have been reported on 30 ornamental plant species in Iran. The most important group reported on ornamental is 16SrII-D, which was associated with *Zinnia elegans*, *Calendula officinalis*, *Phoenix canariensis*, *Petunia violacea*, *Cosmos bipinnatus*, *Conocarpus erecta*, *Albizia lebbeck*, *Tamarix aphylla*, and *Cupressus sempervirens* [160,169,170,171,172]. Some of these are shade trees planted in parks. *Petunia hybrid* was the only ornamental plant found to be associated with the 16SrII-B subgroup [173]. *Austroagallia sinuata* and *O. albicinctus* have been reported as vectors of *Z. elegans* phyllody and petunia witches’ broom disease in Iran [160,174]. In addition, members of the aster yellows phytoplasmas were also associated with other ornamental plants in Iran. For example, *Tagetes patula*, *Gomphocarpus physocarpus*, *Tanacetum partenium*, *Rudbeckia hirta*, *Cereopsis lanceolate*, Gaillardia, and China aster have been identified to be the hosts of the16SrI phytoplasma group [175]. Five host plant species were identified to be associated with 16SrVI, including *Juniperus procumbens*, *Salix babylonica*, *Salix alba*, *Cota tinctoria*, and *Celosia argenta* [176,177,178]. Other host plants, like *Chrysanthemum morifolium*, *Robinia pseudoacacia*, and *Salix alba*, are associated with 16SrIX [179]. 16SrXII phytoplasma strains were associated with *Euonymus japonicas*, *Eucalyptus camaldunensis*, *Narcissus tazetta*, and *Rosa canina* in Iran [180,181,182]. The symptoms include witches’ broom, virescence, early decline, purpling of leaves, phyllody, and virescence.

Seven diverse phytoplasma groups have been reported to be associated with ornamental plants in Israel. These include 16SrVI on *Anemone* sp., *Cosmos* sp., *Lavandula* sp., and *Verbesina encelioides* [183,184], 16SrI and 16SrIII on *Celosia* sp. [185], and 16SrXII on *Cyclamen* sp. and *Lisianthus* sp. [84,86]. *Lymonium* hybrids were reported to be the host of three diverse phytoplasma groups, including 16SrII, 16SrV, and 16SrIX [86]. Periwinkle (*Catharanthus roseus*) is used as test plants and it is cultivated in municipal lands and private garden as an ornamental plant [186]. Diverse phytoplasma strains of 16SrI (Egypt, Iran) [146,175], 16SrII (Saudi Arabia, Egypt) [146,187], 16SrVI (Iran and Turkey) [140,175], and 16SrIX groups [188] have been detected in naturally infected periwinkle. 

Some crassula plant species have been reported developing phytoplasma diseases. For example, *Opuntia abjecta*, *Crassula argentea*, and *Opuntia* sp. were associated with the phytoplasma 16SrII group in Egypt and Lebanon [145,146]. In addition, an association between 16SrII and Arabic jasmine (*Jasminum sambac*) has been reported in Oman and Iraq [62,189]. In Saudi Arabia, the association between 16SrII-D and *Hibiscus rosa-sinensis*, *Calendula officinalis*, *Z. Magellan*, *Plectranthus scutellarioides*, *Conocarpus lanceolatus*, and *Washingtonia robusta* have been reported [190,191]. Moreover, the association between 16SRVI-A and *Washingtonia* sp. has been reported from Kuwait [192].

*Orosius albicinctus* is the insect species identified as a putative vector of *Cota tinctoria* in Iran [176], while *Circulifer orientalis* is a potential vector of phytoplasmas to *Lymonium* in Israel [86].

## 10. Weeds

Weeds can play a role in the survival (alternative hosts) and spread of phytoplasmas. Salehi, Izadpanah, Nejat, and Siampour [150] stated that wild lettuce could be a reservoir of lettuce phyllody phytoplasma in Iran, as they were associated with the same strain and *N. fenestratus*, the confirmed vector, was active in these fields. ‘*Ca*. P. australasia’ strains are known to be associated with many crop diseases, like parsley witches’ broom, tomato witches’ broom, pomegranate little leaf squash, and cucumber phyllody, and ornamental crops, such as petunia witches’ broom [160] and zinnia phyllody [169] in Iran. Hemmati**, et al. [193] reported the association between *Aerva javanica* and 16SrII-B. Given that Mexican lime as well as some other crops, like bell pepper and faba bean, are associated with 16SrII-B, *A. javanica* can act as reservoirs for such an important disease in south of Iran. 

Bermuda grass (*Cynodon dactylon* L.) has been reported to be infected by Bermuda grass white leaf (BGWL) disease, which is attributed to the 16SrXIV group ‘*Ca*. P. cynodontis’ in Iran [194], Turkey [195], Iraq [62], and Saudi Arabia [196]. It has been confirmed that the leafhopper *Exitianus capicola* is the vector of this disease in Iran [194]. The infected plants show bushy growth, whitening of leaves, little leaves, and plant death [197]. Two subgroups of 16SrXIV (A, D) can be differentiated by *Hinf*I restriction enzyme [194]. 

Several weed species in Saudi Arabia and Oman are associated with the 16SrII subgroups [196,198]. The association between 16SrIX and 16SrXXIX has been reported in *Echinops spinosissimus* and *Cassia italica* in Oman [155,199].

## 11. Management of Phytoplasma Diseases

Several management strategies have been developed to manage or reduce the impact of phytoplasma diseases on plant growth and yield. Quarantine is very important in helping to limit the spread of phytoplasmas into areas or countries in which they do not exist [200]. Weed species can work as reservoirs of phytoplasmas and insect vectors can develop their life cycle on them in the absence of hosts [50]. One of the most important methods of managing phytoplasmas is to control insect vectors. The management of pear decline has been achieved through the control of the insect vector *Cacopsylla pyri* [201]. Several other insecticides have been proposed for the management of psyllids transmitting pear decline and apple proliferation phytoplasmas [202]. The use of phytoplasma-free planting material is one of the most important considerations when managing phytoplasma diseases [203]. The injection of tetracycline into coconut palm and elm trees has been found to be effective in reducing phytoplasma diseases in these trees, but it is not cost-effective [204,205]. The development of resistant plant varieties to phytoplasmas is a promising option. Many carrot (*Daucus carota*) varieties showed significant resistance against the aster yellows phytoplasma in the USA and Canada [206]. The recovery of symptomatic plants after infection, especially in apple, stone fruits, and grapevine [207], has been found to produce resistant plants, which was attributed to stimulating jasmonate (JA) and Ca^2+^ signal-related defense mechanisms [208,209]. In addition, the use of resistance stimulants proved to be effective in managing grapevine phytoplasma diseases in Iran [104]. In Oman, WBDL in acid lime is managed through the removal of symptomatic branches, which is anticipated to reduce phytoplasma inoculum level and insect vector attraction, and subsequently delay acid lime death [114,122]. Additionally, environmental conditions have been found to affect WBDL expression, which may indicate that growing acid limes in areas less conducive to phytoplamas could be an important management option [51]. 

## 12. Conclusions

Several phytoplasma diseases have been reported from most countries in the Middle East. Phytoplasmas of the 16SrII group are more distributed in the south of Iran and the Gulf States, while phytoplasmas of the 16SrIX group are distributed in northeast Iran, Turkey, and Lebanon. Differences in the geographical distribution of these phytoplasma diseases, as well as the differences in strains causing the same disease in different countries, could be related to the vectors that are present in certain locations as well as the source of phytoplasmas. One plant species can be a host of diverse or specific strains; however, one strain can infect numerous plant species, indicating that there is no host species-specific system.

Most of the studies in the Middle East focused on the phytoplasma strains associated with different diseases symptoms, with little attention to the vectors of alternative hosts. Therefore, it is important to characterize the vectors of various phytoplasmas in order to develop efficient management programs. In addition, more attempts should be done to identify the role of alternative host species in phytoplasma diseases epidemiology. Further studies should consider: (i) the host-vector interaction and (ii) the role of secondary host species. In addition, limited information is available on the management of phytoplasma diseases, especially in the Middle East. Therefore, it is important to focus future studies on developing integrated management options of phytoplasma diseases.

## Supplementary Materials

The following are available online at https://www.mdpi.com/2079-7737/10/3/226/s1, Table S1: Phytoplasma groups and their distribution, hosts, symptoms and vectors in the Middle East.
