# Supplementary material for: History and Current Status of Phytoplasma Diseases in the Middle East
Source: Biology (Basel). 2021 Mar 15;10(3):226. doi: 10.3390/biology10030226 (PMC8000475; doi:10.3390/biology10030226)
Supplement: Supplementary file 1 [file biology-10-00226-s001.pdf]

Table S1: Phytoplasma groups and their distribution, hosts, symptoms and vectors in the Middle East

Plant	Province/District	Symptoms	16Sr Group/subgroup	Vector status	Detection method	GenBank acc. No.	Reference
**Iran**							
**Fruit crops**							
Almond (*Prunus dulcis*)	Fars, Chaharmahal-O-Bakhtiari, Isfahan	Sever proliferation, little leaves, internode shortening, leaf necrosis, dieback	IX-C<br>II-C<br>XII-A<br>VI-D	*Frutioidea bisignata*	SA*, RFLP	DQ195209<br>DQ195210	(Salehi et al., 2006; Zirak et al., 2009a)
Citrus aurantifolia	Sistan and Baluchestan, Hormozgan, Kerman	Proliferation of axillary shoots, little leaves, yellowing, internode shortening	II-B	*Hishimonus phycitis*	RFLP, EM, TEM, SA	U15442	(Bove and Garnier, 2000)
Prunus scoparia	Fars, Kerman, Kohgiluye and Boyer Ahmad, Isfahan,	Yellowing, witches' broom	IX-B	UK	SA, RFLP	KM235725	(Salehi et al., 2015b)
GF-677*	Fars	Witches' broom, internode shortening, little leaves, proliferation of slender upright shoots, stunting, dieback	IX-B	UK	SA, RFLP	JF781493-4	(Salehi et al., 2011a)

Peach (*Prunus persica*)	Isfahan, Chaharmahal-O-Bakhtiari, Tehran, Kurdestan	Yellowing, resetting, leaf rolling, little leaf, bronzing of foliage	II-C<br>XII-A<br>VI-A<br>IX-D<br>IX-B	UN	SA, RFLP	FJ204393-6	(Zirak et al., 2010b; Salehi et al., 2020)
Russian Olive tree (*Elaeagnus angustifolia*)	Urmia, Azarbaijan Sharghi	Little leaves, axillary shoot proliferation, internode shortening, witches' broom, resetting, dieback	I-A	*Macropsis infuscata*	SA	EU886968	(Rashidi et al., 2010a; Hajizadeh et al., 2017)
Grapevine (*Vitis vinifera*)	Isfahan, Chaharmahal-O-Bakhtiari, Yazd, Markazi, Hamedan, Qazvin, Lorestan, Markazi, Fars	Leaf rolling, leaf yellowing, leaf reddening, decline, thickening and downward rolling of leaves, vein chlorosis, necrosis, shoot dieback, stunting	XII-A<br>II-B<br>VII-A<br>IX-C<br>I-B	*Psammotettix alienus*<br>*Agallia ribauti*	SA, RFLP	KJ637201-7<br>KX267563<br>KX247852<br>KY745782	(Mirchenari et al., 2015; Salehi et al., 2016a; Babaei et al., 2019; Zamharir et al., 2019)
Sweet cherry (*Prunus avium*)	Isfahan, Tehran	Leaf rolling, witches' broom, rosetting	I-B<br>II-B	UN	SA, RFLP	FJ204397-8	(Zirak et al., 2010a)
Plum (*Prunus domestica*)	Tehran, Isfahan, Chaharmaha-O-Bakhtiari, Mazandran	Leaf rolling, little leaf, resetting, yellowing, shoot proliferation	II-B<br>XII-A<br>X-F	UK	SA, RFLP	FJ409624<br>FJ204400<br>KF739403	(Zirak et al., 2009b; Allahverdi et al., 2014)
Apricot (*Prunus armeniaca*)	Fars	Leaf yellowing, inward leaf curl, scorch of leaf margins, shortened	IX-B<br>II-C	UK	SA, RFLP	KY014991-3	(Salehi et al., 2018c; Rasoulpour et al., 2019)

		internodes, rosette, decline, stunting					
Pomegranate (*Punica granatum*)	Fars, Khorasan	Yellowing, little leaf, internode shortening, witches' broom, decline, small and malformed fruits, dried and discolored seeds Redenning and thickening of veins, dieback	II-D<br>III-A	UK	SA, RFLP	KT265695	(Karimi et al., 2015; Salehi et al., 2016b)
Pistachio (*Pistachia vera*)	Qazvin, Kerman, Khorasan Razavi, Qom, Yazd	Sever witches' broom, stunted growth, leaf resetting, leaf narrowing, yellowing, malformation, proliferation	XII-A<br>IX<br>II	UK	SA, FLP	GQ379222<br>MF037208	(Ghayeb Zamharir and Mirabolfathi, 2011; Ghayeb Zamharir, 2018b)
Pear (*Prunus communis*	Fars, Isfahan, Tehran, Khorasan	Grown reduction, leaf curl, abnormal reddening of foliage, defoliation and slow decline	X-C<br>X-B<br>I-B	UN	SA, RFLP	DQ471321<br>KC902810<br>KC902808	(Salehi et al., 2008b; Hashemi-Tameh et al., 2014b)
Apple (*Malus domestica*)	Isfahan, Chaharmahal-O-Bakhtiari	Defoliation, dieback, fruit anomalies, leaf rolling, little leaf, proliferation, resetting, stipule	I<br>II-B	UK	SA, RFLP	KC902794-9	(Hashemi-Tameh et al., 2014a)

		enlargement, yellowing, decline					
Citrus sinensis	Hormozgan, Kerman, Sistan nad Baluchestan	Decline, yellowing	II-B<br>IX	*Diaphorina citri*	SA	MN104092	(Abbasi et al., 2019)
Citrus reticulata	Hormozgan, Kerman	Vein clearing, mottling, leaf yellowing,	II-B	*Hishimonus phycitis*	SA		(Djavaheri and Rahimian, 2004)
Citrus medica	Kerman	Witches' broom	II-B	UN	SA		(Azadvar et al., 2015)
Limequat (*Citrus aurantifolia* × *Fortunella*)	Hormozgan	Witches' broom, little leaf, internode shortening	II-B	UN	SA	KY171947	(Faghihi et al., 2017)
Date palm (*Phoenix dactylifera*)	Khuzestan	Streak yellow leaves	VI-A<br>VII-A	UN	SA	KX374967	(Ghayeb Zamharir and Eslahi, 2019)
Prunus armeniaca	Sistan –O-Baluchestan	rosette	IX-B	UN	SA		(Zamharir and Nazari, 2019)
Chikoo (*Manilkara zapota*)	Hormozgan	Fasciation, little leaves, internode shortening	II-B	UN	SA	KY171945	(Bagheri et al., 2017)
Oleaster (*Elaeagnus angustifolia*)	Qazvin, Azarbaijan Sharghi, Isfahan	Proliferation, witches' broom	II-C	UN	SA	MN877915	(Zamharir and Mohammadipour, 2016)
Barberry (*Berberis vulgaris*)	Khorasan	Stem fasciation, little leaf	II-C	UN	SA, VRFLP	KT879852	(Tavanaei et al., 2016)
**Vegetables and Peas**							
Tomato (*Solanum lycopersicon*)	Bushehr, Fars, Markazi,	Witches' broom, long, curved spindly shoots,	II-D<br>XII-A<br>I-B	UK	SA, RFLP	KC182528	(Salehi and Hosseini, 2016)

	Ardabil, Kurdistan, Khorasan, Azarbaijan Gharbi, Azarbaijan Sharghi, Kermanshah, Urmia	little leaves, big bud, swollen and virescence bud, bushy	VI-A<br>IX-E				
Cabbage (*Brassica olearacea*)	Fars	Stunting, little leaves, yellowing, opening the head, proliferation of the buds, witches' broom	VI-A	*Circulifer haematoceps*	SA, RFLP	DQ195214	(Salehi et al., 2007b)
Potato (*Solanum tuberosum*)	Isfahan, Tehran, Chaharmahal-O-Bakhtiari, Hamedan, East Azerbaijan, Kermanshah, Alborz	Little leaf, leaf rolling, stunting, slight purple top, discoloration of newly growth, swollen node, formation of aerial tubers	VI-A<br>I-B<br>XII-A<br>VI-D	UK	SA, RFLP	FJ427296-7<br>EU649681<br>EU661607	(Hosseini et al., 2011)
Cucumber (*Cucumus sativus*)	Yazd, Kerman, Urmia;<br>Fars,<br>Tehran	Proliferation of short spindly shoot along the stem, little leaves, shortening internodes, fruit cracking, virescence, phyllody, branch	II-M<br>VI-A	*Orosius albicinctus*	SA,RFLP	KR822804<br>GQ861500	(Salehi et al., 2015c; Zibadoost et al., 2016; Esmaeilzadeh-Hosseini et al., 2019)

		malformation, witches' broom					
Squash (*Cucurbita* sp.)	Yazd, Urmia	Proliferation of short spindly shoot along the stem, little leaves, shortening internodes, fruit cracking, virescence, phyllody, branch malformation, witches' broom	II-D<br>VI-A	*Orosius albicinctus*	SA,RFLP	KR822805	(Salehi et al., 2015c; Zibadoost et al., 2016)
Garden beet (*Beta vulgaris*)	Yazd	Narrow leaves grown from crown, internode shortening, excessive growth of axillary buds, stunting, yellowing with gradual reddening of the lower leaves	II-E	*Orosius albicinctus*	SA, RFLP	DQ327722	(Mirzaie et al., 2007)
Spinach (*Spinacia oleracea*)	Kerman	Yellowing, proliferation	I-B	UK	SA, RFLP	GQ861505	(Tazehkand et al., 2010a)
Eggplant (*Solanum melongena*)	Hormozgan	phyllody	IX-C	UK	SA, RFLP	JX464669	(Tohidi et al., 2015)
Onion (*Allium cepa*)	Isfahan	Flower malformation	I-B	UK	SA, RFLP	HM626107	(Sichani et al., 2014)

lettuce (*Lactuca sativa*)	Isfahan	Leaf deformation	I-B<br>IX-B	*Neoaliturus fenetratus*	SA, RFLP	JQ015290	(Salehi et al., 2007a; Sichani et al., 2014)
Carrot (*Daucus carota*)	Yazd, Isfahan	Little leaf, yellowing, proliferation, stunting of taproot, virescence, phyllody, reddening, witches' broom	II-C<br>I-L	*Orosius albicinctus*	SA, RFLP	HQ286477	(Salehi et al., 2016c)
Bell pepper (*Capsicum annum*)	Hormozgan	Yellowing, big bud, little leaf, virescence	II-D	UN	SA	KR706433-4	(Faghihi et al., 2016)
Capsicum frutesens	Urmia	Dwarfing, witches' broom, little leaves	VI-B	UN	SA	KT807466	(Zibadoost et al., 2016)
Red cabbage (*Barssica oleracea*)	Urmia	Little leaf	VI-D		SA	KT807469	
Phaseolus vulgaris	Urmia	Little leaf	VI-A		SA	KT807468	
Faba bean (*Vicia faba*)	Fars, Bushehr	Proliferation of axillary buds from the stem, little leaf, flower virescence, phyllody, proliferation, flower sterility	II-D	*Orosius albicinctus*	SA, RFLP	KU501295	(Salehi et al., 2016e)
Eruca sativa	Yazd	Crown proliferation, witches' broom, little leaf, flower	I-B	UN	SA, VRFLP	KT626568	(Esmailzadeh Hosseini et al., 2015)

		virescence, phyllody, sterility and stunting					
Scallion (*Allium cepa*)	Yazd Isfahan	Flower malformation	I-L	UN	SA, RFLP	HM626109	(Sichani et al., 2014)
Parsley (*Petroselinum crispum*)	Yazd	Development of short spindly shoots from crown buds, little leaf, yellowing, witches' broom, stunting, phyllody and virescence	II-D	UN	SA, RFLP	KU501295	(Salehi et al., 2016d)
Soybean (*Glycine max*)	Golestan, Mazandran	Bud proliferation, aborted seed pods,	VI-B	UN	SA	MG992479-80	(Ghayeb Zamharir and Aldaghi, 2018)
**Ornamental plants**							
Tagetes (*Tagetes patula*)	Markazi	Witches' broom, virescence, early decline, purpuling of leaves	I-B	UK	SA, RFLP	NA	(Babaie et al., 2007a)
Swan plant (*Gomphocarpus physocarpus*)	Markazi	Witches' broom, dwarfing, yellowing and purpling	I-B	UN	SA, RFLP	NA	
Periwinkle (*Cartharanthus roseus*)	Markazi	Yellowing, dwarfing, witches' broom, phyllody	I-A	UN	SA, RFLP	NA	
Tanacetum (*Tanacetum partenium*)	Markazi	Phyllody, stunting	I-B	UN	SA, RFLP	NA	

Cockscomb (*Celosia argenta*)	Markazi	Dwarfing, leaf yellowing, phyllody, dieback	VI-A	UN	SA, RFLP	NA	
Black-eyed Susan (*Rudbeckia hirta*)	Markazi	Phyllody, dwarfing, virescence	I-A	UN	SA, RFLP	NA	
Tickseed (*Cereopsis lanceolate*)	Markazi	Dwarfing, witches' broom, phyllody	I-B				
Aegean wallflower (*Erysium cheiri*)	Kerman	Witches' broom, phyllody	II	UK	SA, RFLP	GQ861503	(Tazehkand et al., 2010b)
Gaillardia	Markazi	phyllody	I-B	UK	SA, RFLP	HQ286479,	(Sichani et al., 2014)
China aster	Markazi	Yellowing and stunting	I-B	UK	SA, RFLP	HQ286480	(Sichani et al., 2014)
Zinnia elegans	Hormozgan	Phyllody, virescence, witches' broom, little leaf, yellowing	II-D	*Austroagalia sinuata*	SA	KY501142	(Hemmati and Nikooei, 2017; 2019a)
Petunia hybrida	Sistan and Baluchestan	Witches' broom, yellowing, little leaf, phyllody, virescence	II-B	UK	SA	JX570935	(Faghihi et al., 2014)
Marigold (*Calendula officinalis*)	Yazd	Little leaves, yellowing, phyllody, virescence, proliferation and sterility of flower,	II-D	UK	SA	KU297202	(Esmailzadeh Hosseini et al., 2011a)

		proliferation of axillary buds, witches’ broom, stunting					
Chrysanthemum morifolium	Markazi	Little leaves, stunting, reddish and chlorotic discoloration	IX-D	UN	SA, RFLP	KC176800	(Ghayeb Zamhari, 2017)
Phoenix canariensis	Khuzestan	Leaf yellowing	II-D	UN	SA, RFLP	KX088466	(Azimi et al., 2016)
Cota tinctoria	Hormozgan	Witches' broom, stunting, twisting of the shoots, little leaf	VI-A	*Orosius albicinctus*	SA	MG569790	(Hemmati et al., 2018)
Petunia violacea	Hormozgan	Witches' broom, little leaves, phyllody, virescence	II-D	*Orosius albicinctus*	SA, VRFLP	MH450237	(Hemmati et al., 2019b)
Mexican aster *Cosmos bipinnatus*	Hormozgan	Phyllody, virescence, little leaf, stunting	II-D	UN	SA	MF186858	(Nikooei et al., 2017)
Rosa canina	Zanjan	Stunting, shortening internode, witches; broom, yellow leaves, little leaves,	XII	UN	SA		(Shahverdi et al., 2016)
Narcissus tazetta	Khuzestan	Proliferation of the petals, virescence, phyllody	XII-A	UN	SA, RFLP	KY315180	(Gholami et al., 2018)
Eucalyptus camaldunensis	Fars, Khozestan	Proliferation of auxiliary buds, yellowing, witches’ broom, reddening	I-B<br>XII-A	UK	SA, VRFLP	KT992689<br>KX685876	(Azimi et al., 2017a; Baghaee-Ravari et al., 2018)

		and reduction in leaves, decline and death					
Black locust (*Robinia pseudoacacia*)	Khorasan, Fars	Yellowing, little leaves,	IX-C<br>II-D	UN	SA, VRFLP	KX553989-92	(Karimzade et al., 2018)
White willow (*Salix alba*)	Alborz, Hamedan	Witches' broom	IX-D<br>VI-A	UN	SA	KX500119<br>AY390261	(Ghayeb Zamhari, 2017; Ghayeb Zamharir, 2018a)
Conocarpus erecta	Khuzestan	Little leaf, leaf roll, stem fasciation	II-D	UN	SA	KX088465	(Azimi et al., 2017b)
Babylon willow (*Salix babylonica*)	Isfahan, Markazi, Yazd, Azarbaijan Gharbi	Witches' broom, proliferation of axillary shoots	XII<br>VI-A	UN	SA	MF185361-2	(Ghayeb Zamharir and Taheri, 2017; Shahryari and Allahverdipour, 2018)
Albizia lebbeck	Kerman	Shoot proliferation, short internode, witches' broom, twig dieback	II-D	UN	SA, VRFLP	MN121115	(Salari and Azadvar, 2019)
Euonymus japonicus	Tehran	Little leaves, bushy growth	XII-A	UN	SA	GQ273961	(Rashidi et al., 2010b)
Juniperus procubens	Golestan	Witches' broom	VI-A	UN	SA, RFLP	MH422510	(Ghayeb Zamharir et al., 2019)
Tamarix aphylla	Yazd	Proliferation of the shoots, shortened internode, little leaf	II-D	UN	SA, RFLP	MF422724	(Esmailzadeh Hosseini et al., 2017)

Cupressus sempervirens	Tehran	Witches' broom, abnormal shoot proliferation, dieback	II-D	UN	SA	KU647632	(Bahari et al., 2016)Bahari et al., 2016
**Oilseed and industrial crops**							
Sesame (*Sesamum indicum*)	Yazd, Fars, Kerman	Witches' broom, phyllody, virescence, yellowing, stem proliferation	II-D<br>II-A<br>VI-A<br>IX-C	*Orosius albicinctus*; *Neoalitrus haematoceps*	SA, RFLP		(Salehi et al., 2017)
Rapeseed (*Brassica napus*)	Fars, Qazvin, Yazd, Isfahan, Kerman	Phyllody and virescence	I-B	*Circulifer haematoceps*	SA, RFLP	FJ594960	(Salehi et al., 2011b; Asghari Tazehkand et al., 2017)
Sunflower (*Helianthus annuus*)	Kerman, Isfahan, Yazd, Fars, Urmia	Witches' broom, little leaves, proliferation of axillary shoot, phyllody	VI<br>II-D	UK	SA, RFLP	GQ861502<br>KJ016231	(Salehi et al., 2009a; Tazehkand et al., 2010a; Salehi et al., 2015a)
Canola (*Brassica napus*)	Kerman	Witches' broom, phyllody, malformation of pistil	I-C	UK	SA, RFLP	GQ861504	(Tazehkand et al., 2010b)
Safflower (*Carthamus tinctorius*)	Fars<br>Yazd	Floral virescence, phyllody, proliferation of axillary buds, little leaf	VI-C	UK	SA, RFLP	DQ88948	(Salehi et al., 2008a)
Hemp (*Cannabis sativa*)	Yazd<br>Fars	Stunting, Yellowing, witches, broom	XII-A<br>IX-C	UK	SA, RFLP	KT245224	(Sichani et al., 2011)

							(Rasoulpour et al., 2017)
**Weeds**							
Bermuda grass (*Cynodon dactelyon*)	Fars	White leaves	XIV-A<br>XIV-B	*Exitianus capicola*	RFLP, SA	EF44488<br>DQ195216	(Salehi et al., 2009b)
Milk weed (*Asclepias curassvica*)	Central	Witches' broom, dwarfing, phyllody	VI-A	UN	SA, RFLP	NA	(Babaie et al., 2007b)
Prickly lettuce (*Lactuca serriola*)	Central, Fars, Isfahan	Apical necrosis, witches' broom, decline, Little leaves, proliferation of buds in the crown, reddening of old leaves	I-B<br>IX-B	*Neoaliturus fenetratus*	SA, RFLP	NA	(Babaie et al., 2007b; Salehi et al., 2007a)
Prosopis farcta	Yazd	Small leaves, shortened internodes, proliferation of axillary buds	II-D	*Orosius albicinctus*	SA, RFLP		(Esmailzadeh Hosseini et al., 2011b)
Cardaria draba	Yazd	Dwarfing, virescence, phyllody, infertility flowers	II-D	*Orosius albicinctus*	SA, RFLP		(Esmailzadeh-Hosseini et al., 2011)
Bidens alba	Hormozgan	Phyllody, virescence, witches' broom, little leaves	IX	UK	SA	KY358007	(Hemmati et al., 2017)
Canadian horseweed	Urmia	Leaf malformation, witches' broom	VI-D	UK	SA, VRFLP	KT807470	(Zibadoost and Rastgou, 2016)

(*Conyza Canadensis*)							
Sophora root (*Sophora alopecuroides*)	Urmia, Tehran, Mazandran	Yellowing and little leaves, stunting	VI-D XII-A IX-C	UN	SA, VRFLP	KT807471, KF263685	(Allahverdi et al., 2017)
Common madder (*Rubia tinctorum*)	Urmia	Little leaf	VI-D	UK	SA, VRFLP	KT870472	(Zibadoost and Rastgou, 2016)
Niger seed (*Guizotia abyssinica*)	Yazd, Isfahan	phyllody	I-L	UN	SA, RFLP	HM626105	(Sichani et al., 2014)
Convolvulus arvensis	Yazd	Witches' broom, dwarfing	XXIX-B XII-Agra	UN	SA, VRFLP		(Esmailzadeh Hosseini et al., 2016b)
Periploca aphylla	Hormozgan	Witches' broom, proliferation, shortening internode, shoot dieback	XXX-A	UN	SA	GQ249159	(Faghihi et al., 2010)
Artemisia sieberi	Hormozgan	Witches' broom, little leaf	II-D	UN	SA, RFLP	MK299844	(Hemmati and Nikooei, 2019b)
Aerva javanica	Hormozgan	Leaf roll, witches' broom, shortening internode	II-D	*Austroagalia sinuata*	SA, VRFLP	MK322505	(Hemmati et al., 2019a)
Cyperus sp.	Hormozgan	Whitening of aerial plant parts, stunting	XI-B	UN	SA	MF136620	(Salehi and Esmailzadeh Hosseini, 2017)
Onobrychis vicifolia	Chaharmahal – O- Bakhtiari	Yellowing, little leaf, reddening of leaflet margin, witches' broom, dwarfing	IX-I	UN	SA, RFLP	KX461906	(Esmailzadeh Hosseini et al., 2016a)

Suaeda aegyptiaca	Hormozgan	Witches' broom, proliferation of axillary shoots, shortening internodes, yellowing, little leaves	VI-A	*Neoaliturus pulcher* (Putative)	SA	KY411138-9	(Askari Seyahooei et al., 2017)
Solanum nigrum	Hormozgan	Witches' broom, little leaves, short internode, yellowing	II-D	UN	SA	GQ866886	(Samavi et al., 2012)
Solanum surrattens	Hormozgan	Witches' broom, little leaves, short internode, yellowing	II-D	UN	SA	GU550504	
Convolvulus arvensis	Fars, Lorestan, Yazd	Little leaves, yellowing, internode shortening, witches' broom, stunting	XII-A	UN	SA, VRLFP	MG010135-7	(Salehi et al., 2018a)
Convolvulus glomeratus	Hormozgan	Witches broom, little leaves	IX-J	*Orosius albicinctus*	SA	MG569789	(Nikooei and Hemmati, 2018)
Traogopogon dubius	Fars	Flower virescence, phyllody, crown proliferation, witches' broom	I-B	UN	SA	KR262955	(Salehi and Salehi, 2015)
Sonchus oleraceus	Yazd	Yellowing, reddening, shortening internodes, flower virescence, phyllody,	I-B	UN	SA	MG652627	(Salehi et al., 2018b)

		proliferation, witches' broom					
Taraxacum officinale	Hamedan	Virescence, phyllody, sterility of flowers, proliferation of axillary buds	XII	UN	SA, RFLP		(Nazarporian et al., 2016)
**Field crops**							
Johnson grass (*Sorghum halepense*)	Urmia	Little leaf	VI-A	UK	SA, VRFLP	KT807469	(Zibadoost and Rastgou, 2016)
Maize (Zea mays)	Urmia	Yellowing, dwarfing	VI-H		SA	KT807467	
White clover (*Trifolium repens*)	Isfahan	Little leaf, leaf reddening	II-C	UN	SA, RFLP	JQ710445	(Hosseini et al., 2013)
Alfalfa (*Medicago sativa*)	Fars,Yazd, Kerman, Sistan and Baluchestan, Bushehr, Khorasan, Hamedan, Isfahan, Urmia	Witches' broom, little leaf, internode shortening, flower virescence, phyllody, proliferation and sterility, leaf yellowing, leaf curling, death	II-D<br>II-C<br>II-A<br>I<br>VI-A<br>XII	*Orosius albicinctus*	SA, RFLP	KT81661	(Salehi et al., 2011c; Esmailzadeh Hosseini et al., 2016c)
**Oman**							
**Fruit crops**							
Citrus aurantifolia	All Oman	Proliferation of axillary shoots, little leaves, yellowing, internode shortening	II-B ‘‘*Candidatus* Phytoplasma aurantifolia’’	*Hishimonus phycitis*	RFLP, SA	U15442	(Bové et al., 1988; Zreik et al., 1995)

**Vegetables and peas**							
Tomato (*Solanum lycopersicon*)	Northern Oman	witches' broom, little leaf, big bud, phyllody and hardened thick stems	II-D	UN	SA	KX358566	(Al-Subhi et al., 2018)
Chickpeas (*Cicer arietinum*)	Northern Oman	Phyllody, yellowing and stunting	II-D	UN	RFLP, SA	KX358573	(Al-Saady et al., 2006; Al-Subhi et al., 2018)
Spinach	Northern Oman	phyllody and shoot proliferation	II-D	UN	SA	KX358567	(Al-Subhi et al., 2018)
Squash	Central Oman	stunting, yellowing plants female flowers showing an abnormal proliferation and phyllody. male flowers showing virescence	II-D	UN	SA	KX358565	(Al-Subhi et al., 2018)
Field pea	Northern Oman	phyllody and shoot proliferation	II-D	UN	SA	KX358571	(Al-Subhi et al., 2018)
Faba bean	Northern Oman	phyllody and shoot proliferation	II-D	UN	SA	KX358570	(Al-Subhi et al., 2018)
Eggplant *(Solanum melongena L.)*	Northern Oman	phyllody and shoot proliferation	II-D	UN	RFLP, SA	KX358572	(Al-Subhi et al., 2011; Al-Subhi et al., 2018)
Forage crops							
Alfalfa	All Oman	Proliferation of shoots, yellowing and reduction of	II-D	UN	RFLP, TEM, SA	AF438413 KX358564	(Khan et al., 2002; Al-Subhi et al., 2018)

		leaf size and stem tillering					
Oilseed crops							
Sesame (*Sesamum indicum*)	Northern Oman	phyllody, virescence, excessive development of short shoots and internodes and little leaves	II-D	UN	RFLP, SA	KX358563	(Al-Sakeiti et al., 2005; Al-Subhi et al., 2018)
Ornamental plants and weeds							
Beach naupaka (*Scaevola taccada*)	Northern Oman	witches' broom, malformation and reduced size of the leaves, short internodes and yellowing	II-D	UN	RFLP, TEM, SA	AB257291	(Al-Zadjali et al., 2012)
Arabian jasmine (*Jasminum sambac*)	Northern Oman	yellowing, die back, reduced leaf size, short internodes and proliferation of axillary shoots, reduced overall size, resulting in a bushy plant	II-D	UN	RFLP, TEM, SA	AB257290	(Al-Zadjali et al., 2007)
Crotalaria aegyptiaca	Northern Oman	witches' broom proliferation of shoots, reduced stem height, and an	II-W	UN	RFLP, TEM, SA	KY872734 KY872735 KY872736 KY872737	(Al-Subhi et al., 2017)

		increased number of leaves					
Polygala mascatense	Northern Oman	Stunted, small leaves, bushy growth and phyllody	II	UN	RFLP	NO	(Livingston et al., 2006)
Radish (*Raphanus sativus*)	Northern Oman	phyllody and virescence	VI	UN	RFLP, SA	NO	(Al-Subhi et al., 2008)
Echinops spinosissimus	Northern Oman	excessive leaf and shoot growth and stunting	IX	UN	SA	NO	(Al-Subhi et al., 2007)
Cassia italica	Northern Oman	witches'-broom, stunted growth and yellowing of leaves	XXIX	UN	RFLP, SA	EF666051	(Al-Saady et al., 2008)
**Saudi Arabia**							
**Fruit crops**							
Lime (*Citrus aurantifolia*)	Al-Hassa, Saudi Arabia	decline	II	UN	RFLP, SA	EU980537	(Alhudaib et al., 2009)
Date palm (*Phoenix dactylifera* L.)	Al-Madinah, Al-Qassim, Al-Hassa, Saudi Arabia	Al-Wijam, Stunting and yellowing increase through the years and yellow streaks appear on the petioles. Fruits and fruit stalk were reduced in size	I<br>II	UN	RFLP, SA	DQ913090<br>MH157916<br>MH157918<br>MH155427<br>MH157915<br>KY622126	(Alhudaib et al., 2007; Alhudaib et al., 2008; Abhary and Al-Baity, 2018; Omar et al., 2018b)
Jojoba (*Simmondsia chinensis*)	Al-Qassim, Saudi Arabia	witches' broom and small leaves	II-D	UN	RFLP, SA	KY581663<br>KY581664	(Omar et al., 2017)
**Vegetables and peas**							

Chenopodium morale L.	Al-Hassa, Saudi Arabia	decline	II	UN	RFLP, SA	EU119389	(Alhudaib et al., 2009)
Eggplants (*Solanum melongena* L.)	Al-Qassim and Riyadh, Saudi Arabia	phyllody, little leaves and witches broom	II-X<br>II-D	UN	RFLP, SA	MK465073<br>MK465072	(Omar et al., 2020)
Cabbage (*Brassica oleracea* L.)	Al-Qassim and Riyadh, Saudi Arabia	multiple heads and deformation of heads	II-X<br>II-D	UN	RFLP, SA	MK465069<br>MK465068	(Omar et al., 2020)
Beetroot (*Beta vulgaris* L.)	Al-Qassim and Riyadh, Saudi Arabia	reddening, stunting and leaves proliferation	II-X	UN	RFLP, SA	MK465074	(Omar et al., 2020)
Celery (*Apium graveolens* L.)	Al-Qassim and Riyadh, Saudi Arabia	stunting and yellowing	II-X<br>II-D	UN	RFLP, SA	MK465071<br>MK465070	(Omar et al., 2020)
Potato (*Solanum tuberosum*)	Al-Qassim, Saudi Arabia	aerial tubers and purple and small leaves	II-X	UN	RFLP, SA	MH423498	(Omar et al., 2018a)
Tomato (*Solanum lycopersicon*)	Alhasa, Eastern Province and Jizan, Saudi Arabia	Phyllody, yellowing, stunting and malformation	II-D	UN	RFLP, SA	KF 017472<br>KF 017473<br>KF 017474<br>KF 017475<br>KF 017476<br>HM584815	(Alhudaib and Rezk, 2011; Alhudaib and Rezk, 2014)
Carrot (*Daucus carota*)	Al-Qassim, Saudi Arabia	Fasciation , phyllody, hairy roots proliferation, yellow and purple leaves	II-D	UN	SA	LN898420<br>LN898421<br>LN898422<br>LN898423	(Omar, 2017)
Onion (*Allium cepa*)	Al-Qassim, Saudi Arabia	Yellow and twisting leaves	II-D	UN	SA	LN898434<br>LN898435<br>LN898436<br>LN898437	(Omar, 2017)

Faba bean (*Vicia faba*)	Al-Qassim, Saudi Arabia	Phyllody	II-D	UN	SA	LN898424 LN898425 LN898426	(Al-Saleh and Amer, 2014; Omar, 2017)
Green mustard (*Brassica juncea*)	Al-Qassim, Saudi Arabia	Stunting and curly leaf edges	II-D	UN	SA	LN898432 LN898433	(Omar, 2017)
Field crop							
Alfalfa (Medicago sativa)	Riyadh and Al-Qassim, Saudi Arabia	Stunting, yellows, witches' broom and phyllody	II-D	UN	SA	LN898427 LN898428 LN898429 LN898430 LN898431 JQ808130 JQ818819 JQ818820 JX646694 JX646695	(AL-Saleh et al., 2014; Omar, 2017)
Ornamental crops and weeds							
Conocarpus *Conocarpus lanceolatus*	Al-Qassim, Saudi Arabia	Witches' broom and little leaves	II-D	UN	SA	LT628541	(Omar and Alsohim, 2016)
Mexican fan palm (*Washingtonia robusta*)	Al-Qassim, Saudi Arabia	Stunting and yellowing increase through the years and yellow streaks appear on the petioles	II	UN	RFLP, SA	MH157917	(Omar et al., 2018b)
Zinnia *Zinnia magellan*	Al-Qassim, Saudi Arabia	Phyllody	II-D	UN	SA	LT628540	(Omar and Alsohim, 2016)

Jazania *Gazania rigens*	Al-Qassim, Saudi Arabia	Phyllody, yellows, proliferation	II-D	UN	SA	LN889977 LN889972	(Omar and Alsohim, 2016)
Hibiscus (*Hibiscus rosa-sinensis*)	Al-Qassim, Saudi Arabia	Witches' broom, leaf rolling, yellows, fasciation	II-D	UN	SA	LN889970	(Omar and Alsohim, 2016)
Albizia (*Albizia lebbek*)	Al-Qassim, Saudi Arabia	Yellows, short internodes	II-B	UN	SA	LN889988 LN889989	(Omar and Alsohim, 2016)
Marigold (*Calendula officinalis*)	Al-Qassim, Saudi Arabia	Phyllody, purple color	II-D	UN	SA	LN889978 LN889979 LN889980	(Omar and Alsohim, 2016)
Coleus (*Plectranthus scutellarioides*)	Al-Qassim, Saudi Arabia	Virescence, fasciation	II-D	UN	SA	LN889975 LN889976	(Omar and Alsohim, 2016)
Periwinkle (*Catharanthus roseus*)	Al-Qassim, Saudi Arabia	Virescence, yellows, witches' broom	II-D II-B	UN	SA	LN889971 LN889984 LN889985 LN889986	(Omar and Alsohim, 2016)
**Weeds**							
Bindweed *Convolvulus arvensis*	Al-Qassim, Saudi Arabia	Little leaves, proliferation, purple color, yellows	II-D	UN	SA	LN889974 LN889982 LN889983 LN889981	(Omar and Alsohim, 2016)
Parkinsonia (*Parkinsonia aculeata*)	Al-Qassim, Saudi Arabia	Witches' broom, yellows, dieback	II-D	UN	SA	LN889973 LN889990	(Omar and Alsohim, 2016)
Chicory *Cichorium intybus*	Al-Qassim, Saudi Arabia	bushy phenotype and stunt	IX-J	UN	RFLP, SA	KY986922	(Pérez-López et al., 2018)
Bermuda grass (*Cynodon dactylon*)	Al-Qassim, Saudi Arabia	whitening of the leaves, shortening of the stolons	XIV-A	UN	RFLP, SA	LT220876 LT220879 LT220880	(Omar, 2016)

Giant reed (*Arundo donax*)	Al-Qassim, Saudi Arabia	variegated leaves, yellows, stunting, little leaves and yellows	XIV-A	UN	RFLP, SA	LT220881	(Omar, 2016)
Cooba (Acacia salicia)	Al-Qassim, Saudi Arabia	variegated leaves, yellows, stunting, little leaves and yellows	XIV-A	UN	RFLP, SA	LT220884	(Omar, 2016)
sand olive (Dodonaea angustifolia)	Al-Qassim, Saudi Arabia	variegated leaves, yellows, stunting, little leaves and yellows	XIV-A	UN	RFLP, SA	LT220882 LT220883	(Omar, 2016)
Plantago lanceolata L.	Al-Hassa, Saudi Arabia	decline	II	UN	RFLP, SA	EU119398	(Alhudaib et al., 2009)
Syria							
Tomato	Syria (Homs)	Twisting, yellowing, reddening of leaves, phyllody, virescence, woody of the stem	VI-A	UN	SA, RFLP		(Khalil et al., 2019)
Grapevine Vitis	Cost	Yellowing, leaf rolling, red coloration	XII VI	UN	SA		(Contaldo et al., 2011)
Sesame	Hama	Phyllody, virescence, witches' broom, formation of dark exudates on floral partsand yellowing, shoot apex fasciation, short internode	UN	*Orosius albicinctus* (Putative)	SA		(Khabbaz et al., 2013)

Iraq							
Vegetables and peas							
Eggplant (Solanum melomgena)	Basra	Witches broom, phyllody, viresence, proliferation, stunting	II-D	UN	SA	KX008308	(Alkuwaiti et al., 2017)
Tomato (*Solanum lycopersicon*)	Al-Zubair Basra	Witches broom, phyllody, viresence, proliferation, stunting	II-D	UN	SA	KU724309	(Alkuwaiti et al., 2017)
Cowpea (*Vigna unguiclata*)	Al-Jadriya Baghdad	Flat stem	XIV-D	UN	SA	MK367419	(Al-Kuwaiti et al., 2019)
Field crops							
Alfalfa (Medicago sativa)	Al-Jadriya Baghdad	Little leaves, internode shortening	II-D	UN	SA	MK367415	(Al-Kuwaiti et al., 2019)
Ornamental crops and weeds							
Sand olive (*Dodanaea viscosa*)	Al-Jadriya Baghdad	Witches' broom	XIV-A	UN	SA	MK367411	(Al-Kuwaiti et al., 2019)
Mallow (*Malva* sp.)	Al-Nassiriya	Witches' broom	II-D	UN	SA	KY284836	(Alkuwaiti et al., 2017)
Bermuda grass	Baghdad	White leaf	XIV-A	UN	SA, VRFLP	KY284842	(Alkuwaiti et al., 2017)
Arabic jasmine (*Jasminum sambac*)	Baghdad	Phyllody and virescence	UN	UN	EM	UA	Al-Kuwaiti et al., 2015

**Egypt**							
**Fruit crops**							
Date palm (*Phoenix dactylifera*)	Al-Giza	Yellow longitudinal streak on leaves and midribs	I	UN	SA	KF826615	(Alkhazindar, 2014)
Mango		Phyllody, virescence,	UN	UN	EM	UA	(El-Banna and El-Deeb, 2001)
Vegetables and peas							
Onion	Al-Gharbia	Streak yellow, twisting	II-D	UN	SA, VRFLP	LT715991	(El-Sisi et al., 2017)
eggplant	Elminia	Malformed leaves, virescence, phyllody	II-D	UN	SA	FR257842	(Omar and Foissac, 2012)
tomato	Al-Sharqiya	Stunting, yellowing, purplish leaves, intense proliferation of lateral buds, hypertrophic calyxes, virescence, little leaves	II-D	Empoasca decipiens	SA	FR822700	(Omar and Foissac, 2012)
Squash	Al-Sharqiya	Stunting and virescence, phyllody,	II-D	UN	SA	FR822704	(Omar and Foissac, 2012)
Sugar beet (*Beta vulgaris*)	Al-Sharqiya	Stunt, chlorotic and necrotic leaves and petioles	UN	UN	EM, PCR	UA	(Shazly et al., 2016)
Oilseed crops							
Sesame		Virescence, phyllody, witches' broom,	UN	UN	EM, PCR	NA	(Hamed et al., 2014)

Field crops							
Sugarcane	Al-Sharghia	Yellow leaves	I-B	UN	SA, RFLP	JN223446	(Elsayed and Boulila, 2014)
Ornamental crops and weeds							
Opuntia objecta	Al-Gharbia	Proliferation, cylindrical of cladodes	II-D	UN	SA, VRFLP	LT715993	(El-Sisi et al., 2017)
Periwinkle	Kafrelsheikh	Yellowing, witches' broom, virescence	I<br>II-D	UN	SA, VRFLP	LT715996	(El-Sisi et al., 2017)
Orbia gigantean		fasciation	II	Un	SA	HG421073	(Omar et al., 2014)
Senecio stapeliiformis		fasciation	II	UN	SA	HG421072	(Omar et al., 2014)
Crassula argintea	Al-Gharbiya	Fascinated leaves with trumpet bell shape	II-D	UN	SA	HG421074	(Dewir et al., 2016)
**Weeds**							
Euphorbia coerulescens		fasciation	II	UN	SA	HG421070	(Omar et al., 2014)
Jordan							
Fruit crops							
peach	Al-Jubiha	Yellow or reddish, rolling upward longitudinally along the mid-vein	I	UN	SA, RFLP		(Anfoka and Fattash, 2004)
grapevine		Yellowing, leaf discoloration and curling, berry shriveling, irregular maturation of wood	XII-A	UN	SA, RFLP	KC835139	(Salem et al., 2013)
Vegetables							

tomato	Al-Mafriq	Leaf proliferation of lateral shoots, hypertrophic calyxes and greening of flower petals	VI-A	UN	SA, RFLP		(Anfoka et al., 2003)
Potato		Leaf reddening, deformed tubers	II-B	UN	SA	MH085230-2	(Salem et al., 2019)
Lebanon							
Fruit crops							
Almond		Early flowering, stunted growth, leaf resetting, die-back, off-season growth, proliferation of slender shoots, witches' broom	IX-B	*Tachycixius viperinus*, *Asymmetrasca decedens*	SA, RFLP,TEM		(Abou-Jawdah et al., 2003; Abou-Jawdah et al., 2014)
Pear		Premature reddening, upward rolling of the leaves, premature defoliation and reduce shoot growth	X-C	UN	SA, RFLP		(Choueiri et al., 2007)
Grapevine		Witches' broom	XII-A	UN	SA, RFLP		(Salar et al., 2007)
Malus domestica		Shortening internodes	IX-C		SA, RFLP	KP851765	(Casati et al., 2016)
Malus sylvestris		Virescence	IX-C		SA, RFLP	KP851766	
Pistacia paaestina		Witches' broom	IX-C		SA, RFLP	KP851768	
Vegetables							

Potato		Heavy proliferation, purplish leaves, hypertrophic calyxes	VI-A	UN	SA, RFLP	AM260488	(Choueiri et al., 2007)
Tomato		Witches broom, little leaves, internode shortening	VI-A	UN	SA, RFLP	AM260489	(Choueiri et al., 2007)
Ornamental crops							
Opuntia monacantha		Stem and shoot proliferation,	II-C	UN	SA, RFLP	AY939815	(Choueiri et al., 2005)
Geranium purpureum		Little leaf	IX-C		SA, RFLP	KP851763	(Casati et al., 2016)
**Weeds**							
Bryonia multiflora		Dwarfing	IX-C	UN	SA, RFLP	KP851762	(Casati et al., 2016)
Inula viscosa		Witches' broom	IX-C		SA, RFLP	KP851764	
Osyris alba		Phyllody	IX-C		SA, RFLP	KP851767	
Rhamus punctate		Virescence	IX-C		SA, RFLP	KP851769	
Scolymus maculatus		Witches' broom	IX-C		SA, RFLP	KP851770	
Sinapis arvensis		Witches' broom	IX-C		SA, RFLP	KP851771	
Solanum nigrum		virescence	IX-C		SA, RFLP	KP851772	
Turkey							
**Fruit crops**							
apple	Ankara	Enlarged stipules, rosette formed	X-A	UN	SA		(Canik and Ertunc, 2007)

		growth of shoots, small fruits,					
Grapevine	Antalia	Sever redness, inward curling of leaves	XII<br>I-B<br>IX	UN	SA	UA	(Canik et al., 2011)
Sweet cherry *(prunus avium*	Usak	Proliferation of the branches, off-season flowering, decline	I-B	UN	SA, RFLP	KF476062	(Çaglayan et al., 2013)
Cereals and forage crops							
maize	Adana	Yellowing, short internodes, small corncobs	XIV-A	UN	SA, RFLP	HE599395	(Çağlar et al., 2019)
Vegetables and peas							
Tomato	Adana	Big bud	XII-A<br>VI-A	UN	SA	UA	(Sertkaya et al., 2007)
Pepper	Adana	stolbur	VI-A	UN	SA	UA	
eggplant	Adana	Little and yellowing leaves	VI-A	UN	SA	UA	
Potato	Uzurum	Stunting, upward leaf rolling with reddish or purplish coloration, chlorosis, swollen nodes, proliferation axillary buds, aerial tubers, early plant decline	XII-A	UN	SA, RFLP	HM485579	(Eroglu et al., 2010)
Oilseed crops							

sesame	Adana	phyllody	VI-A<br>IX-C<br>II-D	*Orosius albicinctus*	SA	UA	(Sertkaya et al., 2007; Catal et al., 2013)
Ornamental crops							
periwinkle	Adana	phyllody	VI-A	UN	SA	UA	(Sertkaya et al., 2007)
Ligustrum avalifolium	Adana	Leaf yellowing, witches' broom, dieback, decline	II-A	UN	SA, VRFLP	HE649494	(Çağlar and Elbeaino, 2013; Çağlar et al., 2013)
**Weeds**							
Corchorus olitorius	Adana	Phyllody, littleleaves, short internodes	II-D	UN	SA, RFLP	KM103728	(Özdemir and Cagirgan, 2015)
Bermuda grass	Adana	Light green to yellow streaks on the leaves, bushy growth and stunting	XIV	UN	SA	HE599391	(Çağlar et al., 2013)
**Israel**							
**Fruit crops**							
Apricot	Neot Smadar		XII-A	UN			(Gera et al., 2011)
Grapevine	North, central, south Golan, Arad		I<br>III<br>XII-A	*Hyalestes obsoletus, Circulifer oreintalis*	SA		(Klein et al., 2001; Orenstein et al., 2001)
Papaya	Kfar Maimon		XII-A	UN	SA		(Gera et al., 2005)
Sweet cherry	Galon		VI	UN	SA		(Weintraub et al., 2007)
**Vegetables**							

Carrot	Beit She'an		I<br>III<br>V	*Circulifer haematoceps, Neoaliturus fenestratus*	SA		(Weintraub and Orenstein, 2004)
pepper	Sharon		XII-A	UN	SA		
Ornamental crops							
Anemone sp.	Galilee		VI	UN	SA		(Weintraub et al., 2007)
Catharanthus roseus	Eilat		VI	UN	SA		
Celosia sp.	Sharon	General yellowing, witches' broom, phyllody	I<br>III	UN	SA		(Tanne et al., 2000)
Cosmos sp.	Sharon		VI	UN	SA		(Weintraub et al., 2007)
Cyclamen sp.	Besor		XII-A	UN	SA		
Gypsophila sp.	Arava	Shoot proliferation, little leaves, yellowing, poor flower set	II	UN	SA		(Gera et al., 2007)
Lavandula sp.	Afula		VI	UN	SA		(Weintraub et al., 2007)
Lisianthus sp.	Besor		XII-A	UN	SA		
Limonium hybrids	Arava	Leaf yellowing, narrow leaves, small and white flower, phyllody	II<br>V<br>IX	*Circulifer oreintalis*	SA		Weintraub et al., 2004
Mirabilis jalapa			II	UN	SA		(Sobolev et al., 2007)
Verbena encelioides	Galilee		VI	UN	SA		(Weintraub et al., 2007)
Mirabilis jalapa	Sharon	Small yellow leaves, distorted flower	II	UN	SA		(Sobolev et al., 2007)
Kuwait							

Date palm (*Phoenix dactylefera*)	Mishref	Lethal yellowing, leaf yellowing, brown leaves	IV-A	UN	EM, PCR, TEM	UA	(Al-Awadhi et al., 2002)
Washingtonia robusta	Mishref	Lethal yellowing, leaf yellowing, brown leaves	IV-A	UN	SA	UA	
**UAE**							
Citrus aurantifolia	All UAE	Proliferation of axillary shoots, little leaves, yellowing, internode shortening	II-B ‘‘*Candidatus* Phytoplasma aurantifolia’’	UN	PCR	NO	(Garnier et al., 1991)
SA: sequence analysis; RFLP: Restriction Fragment Length Polymorphism, VRFLP: Virtual Restriction Fragment Length Polymorphism; EM: Electron Microscopy, TEM: Transmission Electron Microscopy; UN: Unknown							

References

Abbasi, A., Hasanzadeh, N., Zamharir, M.G., and Tohidfar, M. (2019). Identification of a group 16SrIX ‘Candidatus Phytoplasma phoenicium’ phytoplasma associated with sweet orange exhibiting decline symptoms in Iran. *Australasian Plant Disease Notes* 14. doi: 10.1007/s13314-019-0342-9.

Abhary, M.K., and Al-Baity, O.A. (2018). Occurrence and distribution of date palm phytoplasma disease in Al-Madinah region, KSA. *Journal of Taibah University for Science* 12**,** 266-272.

Abou-Jawdah, Y., Abdel Sater, A., Jawhari, M., Sobh, H., Abdul-Nour, H., Bianco, P.A., Molino Lova, M., and Alma, A. (2014). *Asymmetrasca decedens* (Cicadellidae, Typhlocybinae), a natural vector of ' *Candidatus* Phytoplasma phoenicium'. *Annals of Applied Biology* 165**,** 395-403. doi: 10.1111/aab.12144.

Abou-Jawdah, Y., Dakhil, H., El-Mehtar, S., and Lee, I.M. (2003). Almond witches'-broom phytoplasma: A potential threat to almond, peach, and nectarine. *Canadian Journal of Plant Pathology* 25**,** 28-32. doi: 10.1080/07060660309507046.

Al-Awadhi, H.A., Hanif, A., Suleman, P., and Montasser, M.S. (2002). Molecular and microscopical detection of phytoplasma associated with yellowing disease of date palms Phoenix dactylifera L. in Kuwait. *Kuwait Journal of Science and Engineering* 29**,** 87-109.

Al-Kuwaiti, N., Kareem, T., Sadaq, F.H., and Al-Aadhami, L.H. (2019). First report of phytoplasma detection on sand olive, cowpea and alfalfa in Iraq. *Journal of Plant Protection Research* 59**,** 428-431. doi: 10.24425/jppr.2019.129744.

Al-Saady, N.A., Al-Subhi, A.M., Al-Nabhani, A., and Khan, A. (2006). First report of agroup 16 srII phytoplasma infecting chickpea in Oman. *Plant Disease* 90**,** 973.

Al-Saady, N.A., Khan, A.J., Kalari, A., Al-Subhi, A.M., and Bertaccini, A. (2008). Candidatus Phytoplasma omanense associated with witches'-broom of *Cassia italica* (Mill.) Spreng. in Oman. *International Journal of Systematic and Evolutionary Microbiology* 58**,** 461-466.

Al-Sakeiti, M., Al-Subhi, A., Al-Saady, N., and Deadman, M. (2005). First report of witches'-broom disease of sesame (*Sesamum indicum*) in Oman. *Plant Disease* 89**,** 530-530.

Al-Saleh, M., and Amer, M. (2014). Molecular characterization of the 16Sr II group of phytoplasma associated with faba bean (Vicia faba L.) in Saudi Arabia. *The Journal of Animal and Plant Sciences* 24**,** 221-228.

AL-Saleh, M.A., Amer, M.A., AL-Shahwan, I.M., Abdalla, O.A., and Damiri, B.V. (2014). Detection and Molecular Characterization of Alfalfa Witches'-Broom Phytoplasma and its Leafhopper Vector in Riyadh region of Saudi Arabia. *International Journal of Agriculture and Biology* 16.

Al-Subhi, A., Hogenhout, S.A., Al-Yahyai, R.A., and Al-Sadi, A.M. (2017). Classification of a new phytoplasmas subgroup 16SrII-W associated with Crotalaria witches' broom diseases in Oman based on multigene sequence analysis. *BMC Microbiology* 17**,** 221.

Al-Subhi, A.M., Al-Saady, N.A., Al-Habsi, K.A., and Khan, A.J. (Year). "First report of Group 16SrVI Phytoplasma in Radish from Oman", in: *The 6th International Scientific Seminar on Plant Health*).

Al-Subhi, A.M., Al-Saady, N.A., and Khan, A.J. (2007). Molecular characterization of phytoplasma associated with Echinops witches' broom disease. *Bulletin of Insectology* 60**,** 289.

Al-Subhi, A.M., Al-Saady, N.A., Khan, A.J., and Deadman, M.L. (2011). First report of a group 16SrII phytoplasma associated with witches'-broom of Eggplant in Oman. *Plant Disease* 95**,** 360.

Al-Subhi, A.M., Hogenhout, S.A., Al-Yahyai, R.A., and Al-Sadi, A.M. (2018). Detection, identification, and molecular characterization of the 16SrII-D phytoplasmas infecting vegetable and field crops in Oman. *Plant Disease* 102**,** 576-588.

Al-Zadjali, A.D., Al-Sadi, A.M., Deadman, M.L., Okuda, S., Natsuki, T., and Al-Zadjali, T.A. (2012). Detection, identification and molecular characterization of a phytoplasma associated with beach naupaka (*Scaevola taccada*) witches' broom. *Journal of Plant Pathology* 94**,** 379-385.

Al-Zadjali, A.D., Natsuaki, T.N., and Okuda, S. (2007). Detection, identification and molecular characterization of a phytoplasma associated with Arabian jasmine (*Jasminum sambak*) witches' broom in Oman. *Journal of Phytopathology* 155**,** 211-219.

Alhudaib, K., Arocha, Y., Wilson, M., and Jones, P. (2007). Al-Wijam, a new Phytoplasma disease of date palm in Saudi Arabia. *Bulletin of Insectology* 60**,** 285-286.

Alhudaib, K., Arocha, Y., Wilson, M., and Jones, P. (2008). First report of a 16SrI, Candidatus Phytoplasma asteris group phytoplasma associated with a date palm disease in Saudi Arabia. *Plant Pathology* 57**,** 366-366.

Alhudaib, K., Arocha, Y., Wilson, M., and Jones, P. (2009). Molecular identification, potential vectors and alternative hosts of the phytoplasma associated with a lime decline disease in Saudi Arabia. *Crop Protection* 28**,** 13-18.

Alhudaib, K., and Rezk, A. (2011). First report of a phytoplasma associated with a witches' broom disease in tomato in Alhasa, Saudi Arabia. *New Disease Reports* 24**,** 20.

Alhudaib, K., and Rezk, A. (2014). Molecular Characterization of Phytoplasma-associated Disease in Tomato (Lycopersicun esculentum) in Saudi Arabia. *International Journal of Virology* 10**,** 180-191.

Alkhazindar, M. (2014). Detection and molecular identification of aster yellows phytoplasma in date palm in Egypt. *Journal of Phytopathology* 162**,** 621-625. doi: 10.1111/jph.12241.

Alkuwaiti, N.A.S., Kareem, T.A., and Sabier, L.J. (2017). Molecular detection of '*Candidatus* Phytoplasma australasia' and '*Ca*. P. cynodontis' in Iraq. *Agriculture* 63**,** 112-119. doi: 10.1515/agri-2017-0011.

Allahverdi, T., Rahimian, H., and Babaeizad, V. (2014). Prevalence and distribution of peach yellow leaf roll in North of Iran. *Journal of Plant Pathology* 96**,** 603. doi: 10.4454/JPP.V96I3.017.

Allahverdi, T., Rahimian, H., and Rastgou, M. (2017). Molecular identification of Candidatus Phytoplasma spp. associated with Sophora yellow stunt in Iran. *Journal of Plant Protection Research* 57**,** 167-172. doi: 10.1515/jppr-2017-0023.

Anfoka, G.H., and Fattash, I. (2004). Detection and identification of aster yellows (16SrI) phytoplasma in peach trees in Jordan by RFLP analysis of PCR-amplified products (16S rDNAs). *Journal of Phytopathology* 152**,** 210-214. doi: 10.1111/j.1439-0434.2004.00831.x.

Anfoka, G.H., Khalil, A.B., and Fattash, I. (2003). Detection and Molecular Characterization of a Phytoplasma Associated with Big Bud Disease of Tomatoes in Jordan. *Journal of Phytopathology* 151**,** 223-227. doi: 10.1046/j.1439-0434.2003.00709.x.

Asghari Tazehkand, S., Hosseinipour, A., Heydarnejad, J., Rahimian, H., and Massumi, H. (2017). Identification of phytoplasmas associated with sesame phyllody disease in southeastern Iran. *Archives of Phytopathology and Plant Protection* 50**,** 761-775. doi: 10.1080/03235408.2017.1379757.

Askari Seyahooei, M., Hemmati, C., Faghihi, M.M., and Bagheri, A. (2017). First report of a '*Candidatus* Phytoplasma trifolii'-related strain associated with *Suaeda aegyptiaca* and its potential vector in Iran. *Australasian Plant Disease Notes* 12**,** 24. doi: 10.1007/s13314-017-0249-2.

Azadvar, M., Ranjbar, S., Najafinia, M., and Baranwal, V. (2015). First report of natural infection of citron (*Citrus medica* l.) by '*Candidatus* Phytoplasma aurantifolia' in Iran. *Journal of Agricultural Biotechnology* 6**,** 15-22.

Azimi, M., Farokhi-Nejad, R., and Mehrabi-Koushki, M. (2016). First report of a'Candidatus Phytoplasma aurantifolia'-related phytoplasma strain associated with yellowing symptoms on pineapple palm in Iran. *New Dis Rep* 34**,** 4-4.

Azimi, M., Farokhi-Nejad, R., and Mehrabi-Koushki, M. (2017a). First report of a'Candidatus Phytoplasma aurantifolia'-related strain associated with leaf roll symptoms on eucalyptus in Iran. *New Disease Reports* 35**,** 2044-0588.2017.

Azimi, M., Farokhinejad, R., and Mehrabi-Koushki, M. (2017b). First report of Candidatus Phytoplasma aurantifolia (16SrII group) associated with Conocarpus erectus disease in Iran. *Australasian Plant Disease Notes* 12**,** 27. doi: 10.1007/s13314-017-0253-6.

Babaei, G., Esmaeilzadeh-Hosseini, S.A., Eshaghi, R., and Nikbakht, V. (2019). Incidence and molecular characterization of a 16SrI-B phytoplasma strain associated with Vitis vinifera leaf yellowing and reddening in the west of Iran. *Canadian Journal of Plant Pathology* 41**,** 468-474. doi: 10.1080/07060661.2019.1590459.

Babaie, G., Khatabi, B., Bayat, H., Rastgou, M., Hosseini, A., and Salekdeh, G. (2007a). Detection and characterization of phytoplasmas infecting ornamental and weed plants in Iran. *Journal of phytopathology* 155**,** 368-372.

Babaie, G., Khatabi, B., Bayat, H., Rastgou, M., Hosseini, A., and Salekdeh, G.H. (2007b). Detection and characterization of phytoplasmas infecting ornamental and weed plants in Iran. *Journal of Phytopathology* 155**,** 368-372. doi: 10.1111/j.1439-0434.2007.01247.x.

Baghaee-Ravari, S., Jamshidi, E., and Falahati-Rastegar, M. (2018). "Candidatus Phytoplasma solani" associated with Eucalyptus witches' broom in Iran. *Forest Pathology* 48. doi: 10.1111/efp.12394.

Bagheri, A., Faghihi, M.M., Khankahdani, H.H., Seyahooei, M.A., Ghanbari, N., and Sarbijan, S.S. (2017). First report of a phytoplasma associated with sapodilla flattened stem disease in Iran. *Australasian Plant Disease Notes* 12. doi: 10.1007/s13314-017-0248-3.

Bahari, A., Alavi, S., Shams-Bakhsh, M., and Saberi, E. (2016). First report of a 16SrII group related phytoplasma associated with witches' broom of Cupressus sempervirens var. horizontalis in Iran. *New Disease Reports* 34.

Bove, J., and Garnier, M. (2000). Witches' broom disease of lime. *Arab Journal of Plant Protection* 18**,** 148-152.

Bové, J.M., Garnier, M., Mjeni, A.M., and Khayrallah, A. (Year). "Witches' broom disease of small-fruited acid lime trees in Oman: First MLO disease of Citrus", in: *Proceedings of the 10th Conference of the International Organization of Citrus Virologists (IOCV)*: Riverside), 307-309.

Çağlar, B.K., and Elbeaino, T. (2013). A novel phytoplasma associated with witches' broom disease of Ligustrum ovalifolium in Turkey. *European Journal of Plant Pathology* 137**,** 113-117. doi: 10.1007/s10658-013-0222-7.

Çağlar, B.K., Satar, S., Bertaccini, A., and Elbeaino, T. (2019). Detection and seed transmission of Bermudagrass phytoplasma in maize in Turkey. *Journal of Phytopathology* 167**,** 248-255. doi: 10.1111/jph.12792.

Çağlar, B.K., Satar, S., and Elbeaino, T. (2013). Detection and molecular characterization of bermuda grass (*Cynodon dactylon*) white leaf phytoplasma from Turkey. *International Journal of Agriculture and Biology* 15**,** 90-94.

Çaglayan, K., Gazel, M., Küçükgöl, C., Paltrineri, S., Contaldo, N., and Bertaccini, A. (2013). First report of 'candidatus phytoplasma asteris' (group 16sri-b) infecting sweet cherries in Turkey. *Journal of Plant Pathology* 95. doi: 10.4454/JPP.V95I4.021.

Canik, D., and Ertunc, F. (2007). Distribution and molecular characterization of apple proliferation phytoplasma in Turkey. *Bulletin of Insectology* 60**,** 335-336.

Canik, D., Ertunc, F., Paltrinieri, S., Contaldo, N., and Bertaccini, A. (2011). Identification of different phytoplasmas infecting grapevine in Turkey. *Bulletin of Insectology* 64**,** S225-S226.

Casati, P., Quaglino, F., Abou-Jawdah, Y., Picciau, L., Cominetti, A., Tedeschi, R., Jawhari, M., Choueiri, E., Sobh, H., Molino Lova, M., Beyrouthy, M., Alma, A., and Bianco, P.A. (2016). Wild plants could play a role in the spread of diseases associated with phytoplasmas of pigeon pea witches'-broom group (16SrIX). *Journal of Plant Pathology* 98**,** 71-81. doi: 10.4454/JPP.V98I1.026.

Catal, M., Ikten, C., Yol, E., Üstün, R., and Uzun, B. (2013). First report of a 16SrIX group (pigeon pea Witches'-broom) phytoplasma associated with sesame phyllody in Turkey. *Plant Disease* 97**,** 835. doi: 10.1094/PDIS-11-12-1100-PDN.

Choueiri, E., Massad, R., Jreijiri, F., Danet, J.L., Salar, P., Bové, J.M., and Foissac, X. (2005). First Report of a 16SrII Group Phytoplasma Associated with Shoot Proliferation of a Cactus (Opuntia monacantha) in Lebanon. *Plant Disease* 89**,** 1129-1129. doi: 10.1094/PD-89-1129B.

Choueiri, E., Salar, P., Jreijiri, F., El Zammar, S., Massaad, R., Abdul-Nour, H., Bové, J.M., Danet, J.L., and Foissac, X. (2007). Occurrence and distribution of '*Candidatus* Phytoplasma trifolii' associated with diseases of solanaceous crops in Lebanon. *European Journal of Plant Pathology* 118**,** 411-416. doi: 10.1007/s10658-007-9142-8.

Contaldo, N., Soufi, Z., and Bertaccini, A. (2011). Preliminary identification of phytoplasmas associated with grapevine yellows in Syria. *Bulletin of Insectology* 64**,** S217-S218.

Dewir, Y.H., Omar, A.F., Hafez, Y.M., El-Mahrouk, M.E.S., and Mourad, R.Y. (2016). Fasciation in Crassula argentea: molecular identification of phytoplasmas and associated antioxidative capacity. *Phytoparasitica* 44**,** 65-74. doi: 10.1007/s12600-015-0497-7.

Djavaheri, M., and Rahimian, H. (2004). Witches'-broom of bakraee (*Citrus reticulata* hybrid) in iran. *Plant disease* 88**,** 683-683.

El-Banna, O.-H.M., and El-Deeb, S. (2001). First record of phytoplasma associated with malformed mango inflorescences in Egypt. *Egyptian Journal of Phytopathology* 29**,** 101-102.

El-Sisi, Y., Omar, A.F., Sidaros, S.A., and ElSharkawy, M.M. (2017). Characterization of 16SrII-D subgroup associated phytoplasmas in new host plants in Egypt. *Archives of Phytopathology and Plant Protection* 50**,** 504-513. doi: 10.1080/03235408.2017.1336154.

Elsayed, A.I., and Boulila, M. (2014). Molecular Identification and Phylogenetic Analysis of Sugarcane Yellow Leaf Phytoplasma (SCYLP) in Egypt. *Journal of Phytopathology* 162**,** 89-97. doi: 10.1111/jph.12156.

Eroglu, S., Ozbek, H., and Sahin, F. (2010). First Report of Group 16SrXII Phytoplasma Causing Stolbur Disease in Potato Plants in the Eastern and Southern Anatolia Regions of Turkey. *Plant Disease* 94**,** 1374-1374. doi: 10.1094/PDIS-06-10-0439.

Esmaeilzadeh-Hosseini, S.A., Babaei, G., Purmohamadi, S., and Bertaccini, A. (2019). Phytoplasmas infecting greenhouse cucumber in Iran. *Phytopathogenic Mollicutes* 9**,** 31-32. doi: 10.5958/2249-4677.2019.00016.1.

Esmailzadeh-Hosseini, S.A., Salehi, M., and Mirzaie, A. (2011). Alternate hosts of alfalfa witches' broom phytoplasma and winter hosts of its vector orosius albicinctus in Yazd-Iran. *Bulletin of Insectology* 64**,** S247-S248.

Esmailzadeh Hosseini, S., Babaee, G., Salehi, M., Mirchenari, S., and Bertaccini, A. (2016a). First report of a'Candidatus Phytoplasma phoenicium'-related strain (16SrIX-I) associated with yellowing of *Onobrychis viciifolia* in Iran. *New Disease Reports* 34**,** 30-30.

Esmailzadeh Hosseini, S., Salehi, M., Babaie, G., and Ardakani, A.P. (2017). First report of a 16SrII phytoplasma associated with a witches' broom disease of Tamarix aphylla in Iran. *New Disease Reports* 36.

Esmailzadeh Hosseini, S., Salehi, M., Mirchenari, S.M., Contaldo, N., Paltrinieri, S., and Bertaccini, A. (2016b). Occurrence of a ‘Candidatus Phytoplasma omanense’-related strain in bindweed witches’ broom disease in Iran. *Phytopathogenic Mollicutes* 6**,** 87-92.

Esmailzadeh Hosseini, S., Salehi, M., and Salehi, E. (2015). First report of a 16SrI-B subgroup-related phytoplasma associated with *Eruca sativa* phyllody in Iran. *New Dis. Rep* 32**,** 2044-0588.2015.

Esmailzadeh Hosseini, S.A., Khodakaramian, G., Salehi, M., and Bertaccini, A. (2016c). First report of 16SrVI-A and 16SrXII-A phytoplasmas associated with alfalfa witches’ broom disease in Iran. *Journal of Plant Pathology* 98. doi: 10.4454/JPP.V98I2.015.

Esmailzadeh Hosseini, S.A., Salehi, M., Khanchezar, A., and Shamszadeh, M. (2011a). The first report of a phytoplasma associated with pot marigold phyllody in Iran. *Bulletin of Insectology* 64**,** S109-S110.

Esmailzadeh Hosseini, S.A., Salehi, M., and Mirzaie, A. (2011b). Alternate hosts of alfalfa witches' broom phytoplasma and winter hosts of its vector Orosius albicinctus in Yazd-Iran. *Bulletin of Insectology* 64**,** S247-S248.

Faghihi, M., Bagheri, A., Askari Seyahooei, M., Pezhman, A., and Faraji, G. (2017). First report of *a'Candidatus* Phytoplasma aurantifolia'-related strain associated with witches'-broom disease of limequat in Iran. *New Disease Reports* 35**,** 24-24.

Faghihi, M., Siampour, M., Zaeifi, M., Bagheri, A., Salehi, M., and Samavi, S. (2010). First report of a phytoplasma associated with *Periploca aphylla* witches' broom in Iran. *Plant Pathology* 59**,** 400-400.

Faghihi, M., Taghavi, S., Safaei, A., Siampour, M., and Najafabadi, S. (2016). First report of a phytoplasma associated with bell pepper big bud disease in Iran. *New Disease Reports* 33**,** 2044-0588.2016.

Faghihi, M., Taghavi, S., Salehi, M., Sadeghi, M., Samavi, S., and Siampour, M. (2014). Characterisation of a phytoplasma associated with Petunia witches' broom disease in Iran. *New Disease Reports* 30**,** 21.

Garnier, M., Zreik, L., and Bové, J.M. (1991). Witches' broom, a lethal mycoplasmal disease of lime trees in the Sultanate of Oman and the United Arab Emirates. *Plant Disease* 75**,** 546-551.

Gera, A., Maslenin, L., Weintraub, P.G., and Weintraub, M. (2011). Phytoplasma and spiroplasma diseases in open-field crops in Israel. *Bulletin of Insectology* 64**,** S53-S54.

Gera, A., Mawassi, M., Zeidan, M., Spiegel, S., and Bar-Joseph, M. (2005). An isolate of'Candidatus Phytoplasma australiense'group associated with Nivun Haamir dieback disease of papaya in Israel. *Plant pathology* 54.

Gera, A., Weintraub, P.G., Maslenin, L., Spiegel, S., and Zeidan, M. (2007). A new disease causing stunting and shoot proliferation in Gypsophila is associated with phytoplasma. *Bulletin of Insectology* 60**,** 271.

Ghayeb Zamhari, M. (2017). First report of a '*Candidatus* Phytoplasma phoenicium'-related strain (16Sr IX) associated with Salix witches' broom in Iran. *New Disease Reports* 35.

Ghayeb Zamharir, M. (2018a). Association of ‘Candidatus Phytoplasma trifolii’ related strain with white willow proliferation in Iran. *Australasian Plant Disease Notes* 13. doi: 10.1007/s13314-018-0300-y.

Ghayeb Zamharir, M. (2018b). Molecular study of phytoplasmas associated with pistachio yellows in Iran. *Journal of Phytopathology* 166**,** 161-166. doi: 10.1111/jph.12672.

Ghayeb Zamharir, M., and Aldaghi, M. (2018). First report of a'Candidatus Phytoplasma trifolii'-related strain associated with soybean bud proliferation and seed pod abortion in Iran. *New Disease Reports* 37**,** 15.

Ghayeb Zamharir, M., and Eslahi, M.R. (2019). Molecular study of two distinct phytoplasma species associated with streak yellows of date palm in Iran. *Journal of Phytopathology* 167**,** 19-25. doi: 10.1111/jph.12769.

Ghayeb Zamharir, M., and Mirabolfathi, M. (2011). Association of a phytoplasma with pistachio witches' broom disease in Iran. *Journal of Phytopathology* 159**,** 60-62. doi: 10.1111/j.1439-0434.2010.01697.x.

Ghayeb Zamharir, M., Razavi, M., and Rabbaninasab, H. (2019). First report of '*Candidatus* phytoplasma trifolii'-related strain presence in *Juniperus procubens* witches' broom in Iran. *Phytopathogenic Mollicutes* 9**,** 310-313. doi: 10.5958/2249-4677.2019.00130.0.

Ghayeb Zamharir, M., and Taheri, P. (2017). '*Candidatus* Phytoplasma solani' related strain associated with Babylon willow witches' broom in central provinces of Iran. *Australasian Plant Disease Notes* 12**,** 47. doi: 10.1007/s13314-017-0268-z.

Gholami, J., Ranjbar, R., Bahar, M., Choupannejad, R., and Saed Moucheshi, S. (2018). Molecular detection and characterization of a stolbur phytoplasma associated with Narcissus tazetta phyllody in Iran. *Journal of Phytopathology* 166**,** 372-377.

Hajizadeh, A., Khakvar, R., Bashir, N.S., and Zirak, L. (2017). Detection of Russian olive witches'-broom disease and its insect vector in Northwestern Iran. *Journal of Plant Protection Research* 57**,** 309-313. doi: 10.1515/jppr-2017-0028.

Hamed, A.H., El Attar, A.K., and El-Banna, O.H.M. (2014). First record of a phytoplasma associated with faba bean (*Vicia faba* L.) witches'-broom in Egypt. *International Journal of Virology* 10**,** 129-135. doi: 10.3923/ijv.2014.129.135.

Hashemi-Tameh, M., Bahar, M., and Zirak, L. (2014a). 'Candidatus Phytoplasma asteris' and 'Candidatus Phytoplasma aurantifolia', New Phytoplasma Species Infecting Apple Trees in Iran. *Journal of Phytopathology* 162**,** 472-480. doi: 10.1111/jph.12216.

Hashemi-Tameh, M., Bahar, M., and Zirak, L. (2014b). Molecular characterization of phytoplasmas related to apple proliferation and aster yellows groups associated with pear decline disease in Iran. *Journal of Phytopathology* 162**,** 660-669. doi: 10.1111/jph.12245.

Hemmati, C., and Nikooei, M. (2017). Molecular characterization of a Candidatus Phytoplasma aurantifolia-related strain associated with Zinnia elegans phyllody disease in Iran. *Australasian Plant Disease Notes* 12. doi: 10.1007/s13314-017-0234-9.

Hemmati, C., and Nikooei, M. (2019a). Austroagallia sinuata transmission of "Candidatus Phytoplasma aurantifolia" to Zinnia elegans. *Journal of Plant Pathology* 101**,** 1223. doi: 10.1007/s42161-019-00284-9.

Hemmati, C., and Nikooei, M. (2019b). Phytoplasma infection could affect chemical composition of *Artemisia sieberi*. *Plant Pathology Journal* 35**,** 274-279. doi: 10.5423/PPJ.NT.01.2019.0004.

Hemmati, C., Nikooei, M., Bagheri, A., and Faghihi, M. (2017). First report of a '*Candidatus* Phytoplasma phoenicium'-related strain associated with *Bidens alba* phyllody in Iran. *New Disease Reports* 35**,** 8-8.

Hemmati, C., Nikooei, M., and Bertaccini, A. (2019a). Identification and transmission of phytoplasmas and their impact on essential oil composition in Aerva javanica. *3 Biotech* 9. doi: 10.1007/s13205-019-1843-0.

Hemmati, C., Nikooei, M., and Bertaccini, A. (2019b). Identification, occurrence, incidence and transmission of phytoplasma associated with *Petunia violacea* witches' broom in Iran. *Journal of Phytopathology* 167**,** 547-552. doi: 10.1111/jph.12838.

Hemmati, C., Nikooei, M., and Pasalari, H. (2018). Cota tinctoria and Orosius albicinctus: A new plant host and potential insect vector of 'Candidatus Phytoplasma trifolii'. *Australasian Plant Disease Notes* 13. doi: 10.1007/s13314-018-0298-1.

Hosseini, P., Bahar, M., Madani, G., and Zirak, L. (2011). Molecular Characterization of a Phytoplasma Associated with Potato Witches'-broom Disease in Iran. *Journal of Phytopathology* 159**,** 120-123. doi: 10.1111/j.1439-0434.2010.01732.x.

Hosseini, S., Bahar, M., and Zirak, L. (2013). Detection and Identification of a 16SrII Group Phytoplasma Causing Clover Little Leaf Disease in Iran. *Journal of Phytopathology* 161**,** 295-297. doi: 10.1111/jph.12057.

Karimi, M.R., Paltrinieri, S., Contaldo, N., Kamali, H., Sajadinejad, M., Ajami, M.R., and Bertaccini, A. (2015). Phytoplasma detection and identification in declining pomegranate in Iran. *Phytopathogenic Mollicutes* 5**,** 95-99.

Karimzade, M., Siampour, M., Zakiaghl, M., and Mehrvar, M. (2018). Identification and characterization of a phytoplasma associated with black locust yellow disease in two provinces of Iran. *Crop Protection* 110**,** 261-268. doi: 10.1016/j.cropro.2017.05.010.

Khabbaz, S.E., Alnabhan, M., and Arafeh, M. (2013). First report of the natural occurrence of phyllody disease of sesame in Syria. *International Journal of Scientific Research* 2**,** 1-3.

Khalil, H., Yousef, R.N., Girsova, N.V., Bogoutdinov, D.Z., Kastalyeva, T.B., and Aldenkawe, S. (2019). First report of tomato "big bud" disease in syria caused by 'Candidatus phytoplasma trifolii'-related strain. *Plant Disease* 103**,** 578. doi: 10.1094/PDIS-06-18-1057-PDN.

Khan, A.J., Botti, S., Al-Subhi, A.M., Gundersen-Rindal, D.E., and Bertaccini, A. (2002). Molecular identification of a new phytoplasma associated with alfalfa witches` broom in Oman. *Phytopathology* 92**,** 1038-1047.

Klein, M., Weintraub, P., Davidovich, M., Kuznetsova, L., Zahavi, T., Ashanova, A., Orenstein, S., and Tanne, E. (2001). Monitoring phytoplasma-bearing leafhoppers/planthoppers in vineyards in the Golan Heights, Israel. *Journal of Applied Entomology* 125**,** 19-23.

Livingston, S., Al-Azri, M., Al-Saady, N., Al-Subhi, A., and Khan, A. (2006). first report of 16S rDNA II group phytoplasma on Polygala mascatense, a weed in Oman. *Plant Disease* 90**,** 248-248.

Mirchenari, S.M., Massah, A., and Zirak, L. (2015). Bois noir: New phytoplasma disease of grapevine in Iran. *Journal of Plant Protection Research* 55**,** 88-93. doi: 10.1515/jppr-2015-0012.

Mirzaie, A., Esmailzadeh-Hosseini, S., Jafari-Nodoshan, A., and Rahimian, H. (2007). Molecular characterization and potential insect vector of a phytoplasma associated with garden beet witches' broom in Yazd, Iran. *Journal of Phytopathology* 155**,** 198-203.

Nazarporian, L., Esmailzadeh Hosseini, S., Dehghani, A.M., and Salehi, M. (2016). Occurence of Taraxacum officinalis witches' broom disease in Hamedan Province, Iran.

Nikooei, M., and Hemmati, C. (2018). Molecular characterization of a 16SRIX phytoplasma associated with convolvulus glomeratus witches' broom and with an insect vector in Iran. *Journal of Crop Protection* 7**,** 387-393.

Nikooei, M., Hemmati, C., and Bagheri, A. (2017). Association of 'Candidatus Phytoplasma aurantifolia' with Cosmos bipinnatus phyllody disease in Iran. *Journal of Plant Protection Research* 57**,** 314-317. doi: 10.1515/jppr-2017-0037.

Omar, A.F. (2016). Association of 'Candidatus Phytoplasma cynodontis' with Bermuda grass white leaf disease and its new hosts in Qassim province, Saudi Arabia. *Journal of Plant Interactions* 11**,** 101-107.

Omar, A.F. (2017). Detection and molecular characterization of phytoplasmas associated with vegetable and alfalfa crops in Qassim region. *Journal of Plant Interactions* 12**,** 58-66.

Omar, A.F., Aljmhan, K.A., Alsohim, A.S., and Pérez-López, E. (2018a). Potato purple top disease associated with the novel subgroup 16SrII-X phytoplasma. *International journal of systematic and evolutionary microbiology* 68**,** 3678-3682.

Omar, A.F., Alsohim, A., Rehan, M.R., Al-Jamhan, K.A., and Pérez-López, E. (2018b). 16SrII phytoplasma associated with date palm and Mexican fan palm in Saudi Arabia. *Australasian plant disease notes* 13**,** 39.

Omar, A.F., and Alsohim, A.S. (2016). Identification of new plant hosts of 16SrII group phytoplasmas in Saudi Arabia. *Phytopathogenic Mollicutes* 6**,** 71-76.

Omar, A.F., Alsohim, A.S., Dumonceaux, T.J., and Pérez-López, E. (2020). Molecular characterization of'Candidatus Phytoplasma australasia'16SrII subgroups associated with eggplant, cabbage, beetroot, and celery in Saudi Arabia. *Crop Protection* 127**,** 104970.

Omar, A.F., Dewir, Y.H., and El-Mahrouk, M.E. (2014). Molecular identification of phytoplasmas in fasciated cacti and succulent species and associated hormonal perturbation. *Journal of Plant Interactions* 9**,** 632-639. doi: 10.1080/17429145.2014.882421.

Omar, A.F., and Foissac, X. (2012). Occurrence and incidence of phytoplasmas of the 16SrII-D subgroup on solanaceous and cucurbit crops in Egypt. *European Journal of Plant Pathology* 133**,** 353-360. doi: 10.1007/s10658-011-9908-x.

Omar, A.F., Pérez-López, E., Al-Jamhan, K.M., and Dumonceaux, T.J. (2017). First report of a new jojoba (Simmondsia chinensis) Witches'-broom disease in Saudi Arabia and its Association With Infection by a 'Candidatus Phytoplasma australasiae'-related phytoplasma strain. *Plant Dis* 101**,** 1540.

Orenstein, S., Zahavi, T., and Weintraub, P. (2001). Distribution of phytoplasma in grapevines in the Golan Heights, Israel, and development of a new universal primer. *Vitis* 40**,** 219-223.

Özdemir, Z., and Cagirgan, M.I. (2015). Identification and characterization of a phytoplasma disease of jute (*Corchorus olitorius* L.) from south-western Turkey. *Crop Protection* 74**,** 1-8. doi: 10.1016/j.cropro.2015.03.018.

Pérez-López, E., Omar, A.F., Al-Jamhan, K.M., and Dumonceaux, T.J. (2018). Molecular identification and characterization of the new 16SrIX-J and cpn60 UT IX-J phytoplasma subgroup associated with chicory bushy stunt disease in Saudi Arabia. *International journal of systematic and evolutionary microbiology* 68**,** 518-522.

Rashidi, M., Ghosta, Y., and Bahar, M. (2010a). Molecular identification of a phytoplasma associated with Russian olive witches' broom in Iran. *European Journal of Plant Pathology* 127**,** 157-159. doi: 10.1007/s10658-010-9589-x.

Rashidi, M., Habili, N., and Ghasemi, A. (2010b). First report of a stolbur phytoplasma associated with witches' broom of Japanese spindle (Euonymus japonicus). *Plant Pathology* 59.

Rasoulpour, R., Salehi, M., and Bertaccini, A. (2019). Association of a 'Candidatus Phytoplasma aurantifolia'-related strain with apricot showing European stone fruit yellows symptoms in Iran. *3 Biotech* 9**,** 65. doi: 10.1007/s13205-019-1596-9.

Rasoulpour, R., Salehi, M., and Salehi, E. (2017). Detection and partial characterization of a 16SrIX-C phytoplasma associated with hemp witches'-broom in Iran. *Journal of Plant Pathology* 99**,** 219-223. doi: 10.4454/jpp.v99i1.3815.

Salar, P., Choueiri, E., Jreijiri, F., El Zammar, S., Danet, J.L., and Foissac, X. (2007). Phytoplasmas in Lebanon: characterization of '*Candidatus* Phytoplasma pyri' and stolbur phytoplasma respectively associated with pear decline and grapevine "bois noir" diseases. *Bulletin of Insectology* 60**,** 357-358.

Salari, M.R., and Azadvar, M. (2019). First report of a 16SrII-D phytoplasma associated with *Albizia lebbeck* witches' broom disease in Iran. *New Disease Reports* 40**,** 14. doi: 10.5197/j.2044-0588.2019.040.014.

Salehi, E., Salehi, M., Taghavi, S.M., and Izadpanah, K. (2016a). First report of a 16SrIX group (Pigeon pea witches'-broom) phytoplasma associated with grapevine yellows in Iran. *Journal of Plant Pathology* 98. doi: 10.4454/JPP.V98I2.017.

Salehi, M., and Esmailzadeh Hosseini, S.A. (2017). First report of a 16SRXI group phytoplasma ('Candidatus phytoplasma oryzae') associated with Cyperus Spp. white leaf disease in Iran. *Journal of Plant Pathology* 99**,** 809. doi: 10.4454/jpp.v99i3.3971.

Salehi, M., Esmailzadeh Hosseini, S.A., Rasoulpour, R., Salehi, E., and Bertaccini, A. (2016b). Identification of a phytoplasma associated with pomegranate little leaf disease in Iran. *Crop Protection* 87**,** 50-54. doi: 10.1016/j.cropro.2016.04.007.

Salehi, M., Esmailzadeh Hosseini, S.A., Salehi, E., and Bertaccini, A. (2016c). Molecular and biological characterization of a 16SrII phytoplasma associated with carrot witches' broom in Iran. *Journal of Plant Pathology* 98**,** 83-90. doi: 10.4454/JPP.V98I1.039.

Salehi, M., Esmailzadeh Hosseini, S.A., Salehi, E., and Bertaccini, A. (2016d). Occurrence and characterization of a 16SrII-D subgroup phytoplasma associated with parsley witches' broom disease in Iran. *Journal of Phytopathology* 164**,** 996-1002. doi: 10.1111/jph.12520.

Salehi, M., Esmailzadeh Hosseini, S.A., Salehi, E., and Bertaccini, A. (2017). Genetic diversity and vector transmission of phytoplasmas associated with sesame phyllody in Iran. *Folia Microbiologica* 62**,** 99-109. doi: 10.1007/s12223-016-0476-5.

Salehi, M., Esmailzadeh Hosseini, S.A., Salehi, E., and Bertaccini, A. (2018a). Detection and characterisation of phytoplasma strains associated with field bindweed witches’ broom disease in Iran. *Archives of Phytopathology and Plant Protection* 51**,** 803-813. doi: 10.1080/03235408.2018.1490237.

Salehi, M., Esmailzadeh Hosseini, S.A., Salehi, E., Quaglino, F., and Bianco, P.A. (2020). Peach witches’-broom, an emerging disease associated with ‘Candidatus Phytoplasma phoenicium’ and ‘Candidatus Phytoplasma aurantifolia’ in Iran. *Crop Protection* 127. doi: 10.1016/j.cropro.2019.104946.

Salehi, M., Esmailzadeh, S., and Salehi, E. (2015a). Characterisation of a phytoplasma associated with sunflower phyllody in Fars, Isfahan and Yazd provinces of Iran. *New Disease Reports* 31**,** 6-6.

Salehi, M., Haghshenas, F., Khanchezar, A., and Esmailzadeh-Hosseini, S.A. (2011a). Association of 'candidatus phytoplasma phoenicium' with Gf-677 witches' broom in Iran. *Bulletin of Insectology* 64**,** S113-S114.

Salehi, M., and Hosseini, S.A.E. (2016). The first report of a 16SrXII-A phytoplasma associated with tomato big bud disease in Iran. *Journal of Plant Pathology* 98**,** 692. doi: 10.4454/JPP.V98I3.054.

Salehi, M., Hosseini, S.E., and Salehi, E. (2018b). First report of *a'Candidatus* Phytoplasma asteris'-related strain (16SrI-B) associated with *Sonchus oleraceus* (common sowthistle) phyllody disease in Iran. *New Disease Reports* 37.

Salehi, M., Izadpanah, K., and Heydarnejad, J. (2006). Characterization of a new almond witches' broom phytoplasma in Iran. *Journal of Phytopathology* 154**,** 386-391. doi: 10.1111/j.1439-0434.2006.01109.x.

Salehi, M., Izadpanah, K., Nejat, N., and Siampour, M. (2007a). Partial characterization of phytoplasmas associated with lettuce and wild lettuce phyllodies in Iran. *Plant Pathology* 56**,** 669-676. doi: 10.1111/j.1365-3059.2007.01616.x.

Salehi, M., Izadpanah, K., and Siampour, M. (2007b). Characterization of a phytoplasma associated with cabbage yellows in Iran. *Plant Disease* 91**,** 625-630. doi: 10.1094/PDIS-91-5-0625.

Salehi, M., Izadpanah, K., and Siampour, M. (2008a). First report of 'Candidatus phytoplasma trifolii'-related strain associated with safflower phyllody disease in Iran. *Plant Disease* 92**,** 649. doi: 10.1094/PDIS-92-4-0649A.

Salehi, M., Izadpanah, K., and Siampour, M. (2011b). Occurrence, Molecular Characterization and Vector Transmission of a Phytoplasma Associated with Rapeseed Phyllody in Iran. *Journal of Phytopathology* 159**,** 100-105. doi: 10.1111/j.1439-0434.2010.01731.x.

Salehi, M., Izadpanah, K., Siampour, M., and Esmailzadeh-Hosseini, S.A. (2011c). Polyclonal antibodies for the detection and identification of fars alfalfa witches' broom phytoplasma. *Bulletin of Insectology* 64**,** S59-S60.

Salehi, M., Izadpanah, K., Siampour, M., Firouz, R., and Salehi, E. (2009a). Molecular characterization and transmission of safflower phyllody phytoplasma in iran. *Journal of Plant Pathology* 91**,** 453-458.

Salehi, M., Izadpanah, K., Siampour, M., and Taghizadeh, M. (2009b). Molecular characterization and transmission of bermuda grass white leaf phytoplasma in Iran. *Journal of Plant Pathology* 91**,** 655-661.

Salehi, M., Izadpanah, K., Taghayi, S.M., and Rahimian, H. (2008b). Characterization of a phytoplasma associated with pear decline in Iran. *Journal of Phytopathology* 156**,** 493-495. doi: 10.1111/j.1439-0434.2007.01375.x.

Salehi, M., Rasoulpour, R., and Izadpanah, K. (2016e). Molecular characterization, vector identification and partial host range determination of phytoplasmas associated with faba bean phyllody in Iran. *Crop Protection* 89**,** 12-20. doi: 10.1016/j.cropro.2016.06.016.

Salehi, M., and Salehi, E. (2015). First report of *Tragopogon dubius* witches' broom disease associated with a subgroup 16SrI-B phytoplasma in Iran. *New Disease Reports* 32**,** 17-17.

Salehi, M., Salehi, E., Abbasian, M., and Izadpanah, K. (2015b). Wild almond (*Prunus scoparia*), a potential source of almond witches' broom phytoplasma in Iran. *Journal of Plant Pathology* 97**,** 377-381. doi: 10.4454/JPP.V97I2.017.

Salehi, M., Salehi, E., Siampour, M., Quaglino, F., and Bianco, P.A. (2018c). Apricot yellows associated with '*Candidatus* Phytoplasma phoenicium' in Iran. *Phytopathologia Mediterranea* 57**,** 269-283. doi: 10.14601/Phytopathol_Mediterr-22588.

Salehi, M., Siampour, M., Esmailzadeh Hosseini, S.A., and Bertaccini, A. (2015c). Characterization and vector identification of phytoplasmas associated with cucumber and squash phyllody in Iran. *Bulletin of Insectology* 68**,** 311-319.

Salem, N.M., Quaglino, F., Abdeen, A., Casati, P., Bulgari, D., Alma, A., and Bianco, P.A. (2013). First Report of ‘Candidatus Phytoplasma solani’ Strains Associated with Grapevine Bois Noir in Jordan. *Plant Disease* 97**,** 1505-1505. doi: 10.1094/PDIS-04-13-0428-PDN.

Salem, N.M., Tahzima, R., Abdeen, A.O., Bianco, P.A., Massart, S., Goedefroit, T., and De Jonghe, K.D. (2019). First report of ‘*Candidatus* phytoplasma aurantifolia’-related strains infecting potato (*Solanum tuberosum*) in Jordan. *Plant Disease* 103**,** 1406. doi: 10.1094/PDIS-04-18-0705-PDN.

Samavi, S., Faghihi, M., Hasanzadeh, H., Bagheri, A., Salehi, M., and Sotoudehnia, P. (2012). First report of the natural occurrence of group 16SrII '*Candidatus* Phytoplasma aurantifolia' in two Solanum species in Iran. *New Disease Reports* 26**,** 23.

Sertkaya, G., Martini, M., Musetti, R., and Osler, R. (2007). Detection and molecular characterization of phytoplasmas infecting sesame and solanaceous croos in Turkey. *Bulletin of Insectology* 60**,** 141-142.

Shahryari, F., and Allahverdipour, T. (2018). “Candidatus Phytoplasma trifolii” related strain affecting Salix babylonica in Iran. *Australasian Plant Disease Notes* 13. doi: 10.1007/s13314-018-0321-6.

Shahverdi, S., Afsharifar, A., Taghavi, M., and Behjatnia, S.A.A. (2016). The first report of association of a 16SrXII phytoplasma with witches' broom disease of dog-rose in Iran. *Iranian Plant Protection Congress* 22**,** 92.

Shazly, M., Abagy, E., Aly, A.M., and Youssef, S.A. (2016). Identification and molecular characterization of little leaf disease associated with Phytoplasma on sugar beet (Beta Vulgaris L.) plants in Egypt. *Middle East J Appl Sci* 6**,** 1054-1065.

Sichani, F.V., Bahar, M., and Zirak, L. (2011). Characterization of stolbur (16SrXII) group phytoplasmas associated with *Cannabis sativa* witches'-broom disease in Iran. *Plant Pathology Journal* 10**,** 161-167. doi: 10.3923/ppj.2011.161.167.

Sichani, F.V., Bahar, M., and Zirak, L. (2014). Characterization of phytoplasmas related to aster yellows group infecting annual plants in iran, based on the studies of 16s rRNA and rp genes. *Journal of Plant Protection Research* 54**,** 1-8. doi: 10.2478/jppr-2014-0001.

Sobolev, I., Weintraub, P.G., Gera, A., Tam, Y., and Spiegel, S. (2007). Phytoplasma infection in the four o'clock flower (Mirabilis jalapa). *Bulletin of Insectology* 60**,** 281.

Tanne, E., Kuznetsova, L., Cohen, J., Alexandrova, S., and Gera, A. (2000). Phytoplasmas as causal agents of Celosia disease in Israel. *HortScience* 35**,** 1103-1106.

Tavanaei, S.R., Shams Bakhsh, M., and Akbari Motlagh, M. (2016). The first report pf a phytoplasma associated with Barberry (Berberis vulgaris) stem fasciation in Iran

Tazehkand, S.A., Pour, A.H., Heydarnejad, J., Varsani, A., and Massumi, H. (2010a). Identification of phytoplasmas associated with cultivated and ornamental plants in Kerman province, Iran. *Journal of Phytopathology* 158**,** 713-720.

Tazehkand, S.A., Pour, A.H., Heydarnejad, J., Varsani, A., and Massumi, H. (2010b). Identification of phytoplasmas associated with cultivated and ornamental plants in Kerman province, Iran. *Journal of Phytopathology* 158**,** 713-720. doi: 10.1111/j.1439-0434.2010.01682.x.

Tohidi, Z., Salehi, M., Ghasemi, S., Khanchezar, A., and Shahamiri, S.M. (2015). Association of a 16SrIX-C phytoplasma with eggplant phyllody in Iran. *Journal of Crop Protection* 4**,** 247-256.

Weintraub, P.G., and Orenstein, S. (2004). Potential leafhopper vectors of phytoplasma in carrots. *International Journal of Tropical Insect Science* 24**,** 228-235.

Weintraub, P.G., Zeidan, M., Spiegel, S., and Gera, A. (2007). Diversity of the known phytoplasmas in Israel. *Bulletin of insectology* 60**,** 143.

Zamharir, M.G., and Mohammadipour, M. (2016). Detection and characterization of a 'Candidatus Phytoplasma aurantifolia'-related strain associated with Elaeagnus angustifolia proliferation in Iran. *Phytopathogenic Mollicutes* 6**,** 99-101.

Zamharir, M.G., Mozaffarian, F., and Hosseini-Gharalari, A. (2019). Molecular detection of grape decline phytoplasma in leafhopper species associated with infected grapevines in Iran. *Acta Phytopathologica et Entomologica Hungarica* 54**,** 25-34. doi: 10.1556/038.54.2019.003.

Zamharir, M.G., and Nazari, O. (2019). Identification of 16srIX-B phytoplasmas associated with apricot rosette in Iran. *Phytopathogenic Mollicutes* 9**,** 219-220. doi: 10.5958/2249-4677.2019.00110.5.

Zibadoost, S., and Rastgou, M. (2016). Molecular identification of phytoplasmas associated with some weeds in west Azarbaijan Province, Iran. *Acta Agriculturae Slovenica* 107**,** 129-136. doi: 10.14720/aas.2016.107.1.13.

Zibadoost, S., Rastgou, M., and Tazehkand, S.A. (2016). Detection and molecular identification of ‘Candidatus phytoplasma trifoli’ infecting some cultivated crops and vegetables in West Azarbaijan province, Iran. *Australasian Plant Disease Notes* 11**,** 1-4. doi: 10.1007/s13314-015-0188-8.

Zirak, L., Bahar, M., and Ahoonmanesh, A. (2009a). Characterization of phytoplasmas associated with almond diseases in Iran. *Journal of Phytopathology* 157**,** 736-741. doi: 10.1111/j.1439-0434.2009.01567.x.

Zirak, L., Bahar, M., and Ahoonmanesh, A. (2009b). Molecular characterization of phytoplasmas related to peanut witches' broom and stolbur groups infecting plum in Iran. *Journal of Plant Pathology* 91**,** 713-716.

Zirak, L., Bahar, M., and Ahoonmanesh, A. (2010a). Characterization of phytoplasmas related to 'Candidatus phytoplasma asteris' and peanut WB group associated with sweet cherry diseases in Iran. *Journal of Phytopathology* 158**,** 63-65. doi: 10.1111/j.1439-0434.2009.01566.x.

Zirak, L., Bahar, M., and Ahoonmanesh, A. (2010b). Molecular characterization of phytoplasmas associated with peach diseases in Iran. *Journal of Phytopathology* 158**,** 105-110. doi: 10.1111/j.1439-0434.2009.01585.x.

Zreik, L., Carle, P., Bové, J.M., and Garnier, M. (1995). Characterization of the Mycoplasma like Organism Associated with Witches'-Broom Disease of Lime and Proposition of a Candidatus Taxon for the Organism, "*Candidatus* Phytoplasma aurantifolia". *International Journal of Systematic Bacteriology* 45**,** 449-453.
